# Genetic Dissection of *Anopheles gambiae* Gut Epithelial Responses to *Serratia marcescens*

**DOI:** 10.1371/journal.ppat.1003897

**Published:** 2014-03-06

**Authors:** Stavros Stathopoulos, Daniel E. Neafsey, Mara K. N. Lawniczak, Marc A. T. Muskavitch, George K. Christophides

**Affiliations:** 1 Department of Life Sciences, Imperial College London, London, United Kingdom; 2 Broad Institute, Cambridge, Massachusetts, United States of America; 3 Boston College, Chestnut Hill, Massachusetts, United States of America; 4 The Cyprus Institute, Nicosia, Cyprus; Stanford University, United States of America

## Abstract

Genetic variation in the mosquito *Anopheles gambiae* profoundly influences its ability to transmit malaria. Mosquito gut bacteria are shown to influence the outcome of infections with *Plasmodium* parasites and are also thought to exert a strong drive on genetic variation through natural selection; however, a link between antibacterial effects and genetic variation is yet to emerge. Here, we combined SNP genotyping and expression profiling with phenotypic analyses of candidate genes by RNAi-mediated silencing and 454 pyrosequencing to investigate this intricate biological system. We identified 138 *An. gambiae* genes to be genetically associated with the outcome of *Serratia marcescens* infection, including the peptidoglycan recognition receptor *PGRPLC* that triggers activation of the antibacterial IMD/REL2 pathway and the epidermal growth factor receptor *EGFR*. Silencing of three genes encoding type III fibronectin domain proteins (*FN3Ds*) increased the *Serratia* load and altered the gut microbiota composition in favor of *Enterobacteriaceae*. These data suggest that natural genetic variation in immune-related genes can shape the bacterial population structure of the mosquito gut with high specificity. Importantly, *FN3D2* encodes a homolog of the hypervariable pattern recognition receptor Dscam, suggesting that pathogen-specific recognition may involve a broader family of immune factors. Additionally, we showed that silencing the gene encoding the gustatory receptor Gr9 that is also associated with the *Serratia* infection phenotype drastically increased *Serratia* levels. The Gr9 antibacterial activity appears to be related to mosquito feeding behavior and to mostly rely on changes of neuropeptide F expression, together suggesting a behavioral immune response following *Serratia* infection. Our findings reveal that the mosquito response to oral *Serratia* infection comprises both an epithelial and a behavioral immune component.

## Author Summary

In malaria vector mosquitoes, the presence of bacteria and malaria parasites is tightly linked. Bacteria that are part of the mosquito gut ecosystem are critical modulators of the immune response elicited during infection with malaria parasites. Furthermore, responses against oral bacterial infections can affect malaria parasites. Here, we combined mosquito gut infections with the enterobacterium *Serratia marcescens* with genome-wide discovery and phenotypic analysis of genes involved in antibacterial responses to characterize molecular processes that control gut bacterial infections thus possibly affecting the mosquito susceptibility to infection by malaria parasites. Our data reveal complex genetic networks controlling the gut bacterial infection load and ecosystem homeostasis. These networks appear to exhibit much higher specificity toward specific classes of bacteria than previously thought and include behavioral response circuits involved in antibacterial immunity.

## Introduction

Genetic variation within populations of the *An. gambiae* mosquito, especially with regard to genes encoding immune factors, is believed to play an important role in the mosquito susceptibility to infection by the malaria parasite *Plasmodium falciparum* [1]–[3]. Many immune factors exhibit both anti-*Plasmodium* and antibacterial activities, such as those involved in the IMD/REL2 pathway, which is triggered by bacteria through the peptidoglycan recognition receptor PGRPLC [4], [5]. Bacterial infections can affect mosquito survival [6] and are thought to constitute a major evolutionary drive [7] as opposed to *Plasmodium* infections the impact of which on mosquito fitness is unclear [8]. An example is the segregation of *TEP1* alleles between the M and S molecular forms of *An. gambiae* in west Africa, which differentially affect *Plasmodium* infections, and is thought to be largely driven by bacterial pathogen pressure in larval habitats [2]. Therefore, genetic associations related to the outcome of bacterial infections may, directly or indirectly, influence mosquito vectorial capacity.

The adult mosquito gut harbors a wide spectrum of bacterial populations, mainly Gram-negative enterobacteria [9]–[11]. The broad variation in gut microbiota composition observed both at the individual and population levels is probably the result of an interplay between the environmental bacterial diversity and the mosquito genetic makeup [12]–[14]. Moreover, a precipitous bacterial increase after a blood meal, whose peak coincides with midgut invasion by *Plasmodium* [15], can affect the *Plasmodium* infection load both indirectly, by triggering PGRPLC-mediated mosquito immune responses [5], [16] or through generation of immune memory [17], and directly, through the generation of reactive oxygen species by specific enterobacteria that compromise malaria parasites [18].

Epithelial responses against Gram-negative bacteria have been extensively studied in *Drosophila* [19]. They involve recognition of peptidoglycan [20], [21] that triggers a finely-tuned immune response mainly through the Imd pathway, resulting in the expression of antimicrobial peptides that limit bacterial populations [22], [23]. Production of reactive oxygen species, which target bacteria, through the Dual Oxidase (DUOX) pathway, has also been reported [24]. Gut stem cell proliferation and epithelial cell renewal following tissue damage due to bacterial infection are regulated by the EGFR and JAK/STAT pathways [25]–[27]. However, the mechanisms involved in achieving gut homeostasis remain poorly understood. It has been suggested that regulation of Imd responses can influence the microbiota composition in *Drosophila* [28]. Further discrimination between commensal and pathogenic bacteria can be provided by recognition of pathogen-derived uracil, most likely by unidentified G protein-coupled receptors (GPCRs), which triggers the DUOX pathway [29], but the possibility of more specific responses that shape the gut microbiota remains open.

One unexplored aspect of antibacterial immunity is the behavioral immune responses that limit or disrupt the intake of pathogens, thus making an infection more controllable by the immune system. Feeding behavior in *Drosophila* is known to be finely regulated through an interplay between allatostatin A and neuropeptide F (NPF) [30] while feeding suppression is shown to occur following an immune challenge [31]–[33]. Gustatory receptors are shown to modulate feeding behavior by acting as nutrient sensors [34] and may also be involved in aversion circuits [35] or antibacterial responses through recognition of bacterial-derived metabolites as in mammalian chemoattractant receptors [36].

Here we set out to examine the genetic basis of bacterial infection in the mosquito gut using *An. gambiae* infections with the Gram-negative enterobacterium *Serratia marcescens* that is prevalent in both lab-reared and field collected mosquitoes and is shown to affect the *Plasmodium* infection load [10], [12], [37]. To achieve this, we used an Affymetrix 400 k SNP genotyping array to identify genetic variation associated with the outcome of oral *S. marcescens* infection in a recently established M form *An. gambiae* colony. The results identify 138 genes associated with the outcome of infection, including the gene encoding the major IMD/REL2 receptor PGRPLC and the epidermal growth factor receptor EGFR, and further suggest that epithelial immune responses against gut bacteria are more complex than previously thought. We identify a set of three type III fibronectins that modulate homeostasis of the gut microbiota with specificity mainly against *Enterobacteriaceae*. We also present evidence that behavioral responses following *S. marcescens* infection can modulate the bacterial load. These data could be further exploited in mosquito microbiota-based interventions aiming to limit malaria transmission.

## Results

### *S. marcescens* infection of the mosquito gut

**Figure 1:**
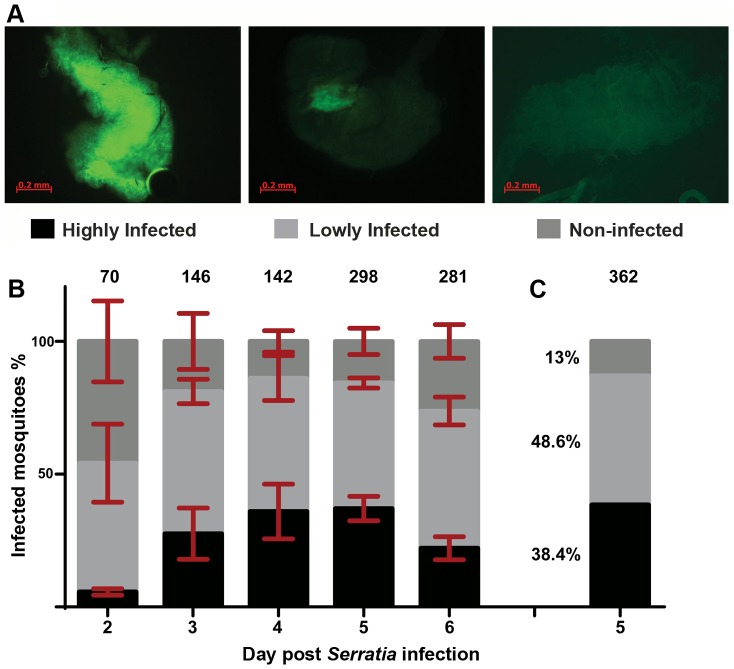
Gut infection with *S. marcescens* varies between individual *An. gambiae* mosquitoes. Mosquitoes were antibiotic treated for 5 days and subsequently fed on sugar containing the Db11-GFP strain of *S. marcescens*. Bacteria-fed mosquitoes were selected 2 days post infection and the prevalence of fluorescent bacteria in their gut was monitored from day 2 to 6 post infection. **1A:** The level of *S. marcescens* infection in the mosquito gut showed considerable variation: mosquitoes with intense fluorescence in most of the gut were characterized as highly infected (left panel), mosquitoes in which fluorescence was evident but confined to a part of the gut were characterized as lowly infected (middle panel) and mosquitoes with no sign of fluorescence were characterized as non-infected (right panel). **1B:** *S. marcescens* infected mosquitoes were dissected each day, from day 2 to 6 post infection, and the proportions of highly, lowly and non-infected mosquitoes were determined over 4 independent infections. The average percentage ±SEM for each level of infection is indicated for each day post infection, with the total number of mosquitoes dissected each day in all 4 infections shown over each bar. **1C:** In 2 independent infections used for SNP genotyping, mosquitoes were dissected 5 days post infection and the percentage of highly, lowly and non-infected mosquitoes, pooled from both infections, can be seen beside the respective part of the bar representing each level of infection.

*An. gambiae* female adults were treated with antibiotics to reduce their natural gut microbiota load (Figure S1) and subsequently fed with fluorescently labeled *S. marcescens* (Db11-GFP) added to the sugar meal. The bacterial levels in the gut of sugar-fed mosquitoes (henceforth referred to as infection) were monitored from day 2 to 6 post infection and showed considerable variation including highly and lowly infected mosquitoes as well as mosquitoes that despite ingesting bacteria-containing sugar showed no sign of fluorescence in their gut (Figure 1A). While the proportion of lowly infected mosquitoes remained rather constant at approximately 50% throughout the course of the experiment, the relative proportions of highly and non-infected mosquitoes changed between days 2 and 3 in favor of highly infected mosquitoes and remained stable thereafter until day 5 (Figure 1B). At day 6, highly infected mosquitoes decreased by ca. 15% with a parallel increase of non-infected mosquitoes.

### Identification of SNP divergence associated with the outcome of *S. marcescens* infection

To investigate whether genetic variation could partly explain the observed *S. marcescens* infection phenotype, single nucleotide polymorphism (SNP) divergence between the highly and non-infected phenotypic pools was interrogated using a 400 k SNP genotyping array. Mosquitoes were orally infected with *S. marcescens*, and gut infection levels were determined at day 5 post infection. The results were similar to those obtained in the previous replicate experiments: 38.4% of mosquitoes could be classified as highly infected, 48.6% lowly infected and 13% non-infected (Figure 1C). Pools of equimolar amounts of genomic DNA (gDNA) prepared from carcasses of 15 highly infected and 15 non-infected mosquitoes out of 139 and 47 mosquitoes in each phenotypic group, respectively, were hybridized onto two Affymetrix SNP genotyping arrays. These SNP chips interrogate genetic variation at ∼400,000 variable positions in the *An. gambiae* genome (Table S1) [38], and were previously shown to provide useful quantitative information regarding divergence between pooled mosquito samples [39].

Allele calls for each SNP locus were used to determine the minor allele frequency (MAF) differences between highly and non-infected gDNA pools. Two approaches were used to assess genotypic association with the *S. marcescens* infection phenotype. The first included MAF difference at a SNP locus between highly infected and non-infected pools >0.5, suggesting a preponderance of different genotypes between the two pools for the respective locus. The second involved a permutation analysis in which the average MAF difference of 10 adjacent SNP loci (SNPs) was compared with that of 10 random SNPs. Statistical significance was assessed for each of the ∼40,000 non-overlapping 10-SNP windows (Table S2) and those showing a p-value<10^−5^, following a Bonferroni correction for the number of tests conducted, were considered as being associated with the *S. marcescens* infection phenotype.

**Figure 2:**
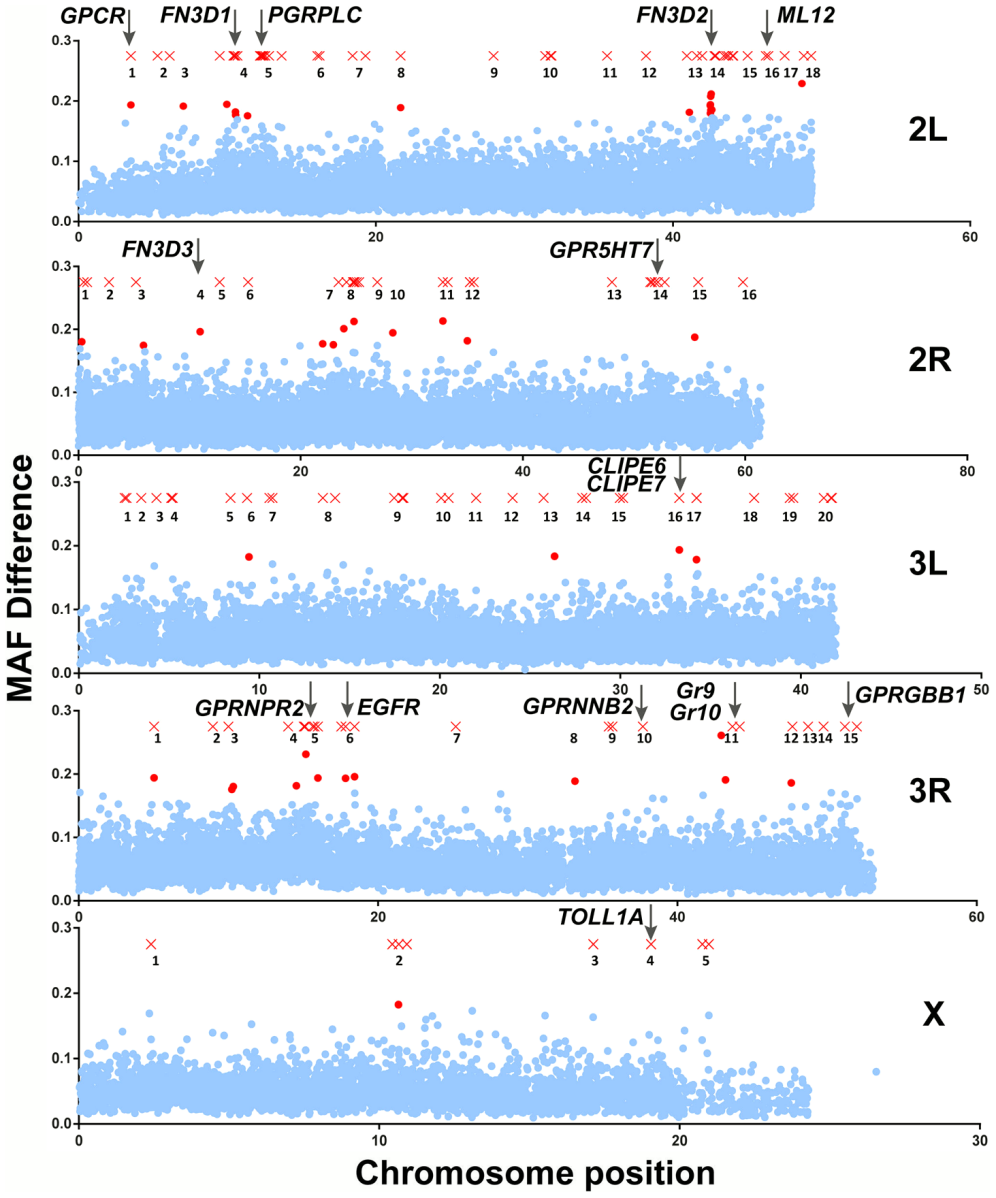
Mapping of *An. gambiae* genetic variation associated with the *S. marcescens* infection phenotype. SNPs with MAF difference >0.5 and 10-SNP windows with Bonferroni-corrected significance (p-value<10^−5^) are shown in their respective chromosomal position as red X crosses and dots, respectively. Non-significant 10-SNP windows are shown as blue dots. Genomic areas with highlighted SNPs and/or significant 10-SNP windows in close proximity are referred to as peaks and are numbered. Each peak is referred to using the chromosomal arms it resides on and its respective assigned number. The genomic positions of genes of interest found within a 5 kb radius of highlighted SNPs or within genomic areas delineated by 10-SNP windows with a significant p-value are indicated by vertical arrows.

The two approaches detected 140 SNPs with MAF difference >0.5 and 44 10-SNP windows with significant p-values, respectively. As shown in Figure 2, these SNPs and 10-SNP windows together formed distinctive clusters along the *An. gambiae* genome that were designated as peaks so that they are discerned from each other, although assessed association was limited to genes within a 5 kb radius of highlighted SNPs or within genomic areas delineated by significant 10-SNP windows. Overall, 118 genes were found to reside within a 5 kb radius of highlighted SNPs (Table S3), while 27 genes fell within significant 10-SNP windows (Table S4). The two approaches combined detected 138 genes (Table S5), as there was an overlap of 7 genes between the two sets, including the highly relevant *CLIPE6* and *EGFR* as discussed below.

In peak 2L-5 (chromosome 2L, peak 5), the gene encoding PGRPLC is found within a 5 kb radius of a highlighted SNP. PGRPLC recognizes peptidoglycan and activates the IMD/REL2 NF-kappaB signaling pathway, thus eliciting antibacterial responses [21], [40], [41]. This pathway is constitutively triggered by mosquito gut bacteria maintaining an elevated level of antimicrobial peptide production [5], [42]. The association of *PGRPLC* with the *S. marcescens* infection phenotype suggests that genetic variation within the mosquito population may influence the ability to mount an antibacterial response via the IMD/REL2 pathway. Adjacent to *PGRPLC*, in peak 2L-5, is another peptidoglycan recognition protein encoding gene, *PGRPLA*.

Of the remaining genes, several exhibit homologies suggesting involvement in antibacterial immune responses, especially in recognition of pathogen or host derived signals as well as in signal transduction and regulation of immune responses (Table 1). The permutation analysis revealed 3 genes, out of a total of 27, encoding proteins with type III fibronectin domains (FN3D) in different peaks: *FN3D1* in peak 2L-4, which was also in the proximity of a highlighted SNP, *FN3D2* in 2L-14 and *FN3D3* in 2R-4. A total of 65 *An. gambiae* genes contain FN3 domains, including the hypervariable pattern recognition receptor *AgDscam*, the insulin receptor *INR* and the JAK/STAT receptor *DOME*. *FN3D2* and *FN3D3* additionally possess immunoglobulin and putative transmembrane domains, while *FN3D2* is an ortholog of *Drosophila Dscam4*. *Drosophila* Dscam is shown to bind bacteria and influence the efficiency of phagocytosis [43], while its *An. gambiae* ortholog, AgDscam, is also shown to bind bacteria and mediate antibacterial and anti-*Plasmodium* responses [44]. Importantly, *Dscam* genes in various organisms generate a diverse repertoire of isoforms, suggestive of challenge-specific pattern recognition through alternative splicing [43], [45], [46], with particular *AgDscam* isoforms specifically targeting *P. berghei*, *P. falciparum* or commensal bacteria [47], [48].

**Table 1:** Genes of interest associated with the *S. marcescens* infection phenotype.

Gene ID	Name/Description	SNP/Permutation analysis	Peak (Figure 2)
**AGAP001111**	alpha-glucosidase	Permutation	2R-1
**AGAP004032**	alpha-mannosidase	SNP	2R-13
**AGAP011785**	CLIPE6	SNP, Permutation	3L-16
**AGAP011786**	*CLIPE7*	SNP, Permutation	3L-16
**AGAP002492**	DNA-binding	Permutation	2R-7
**AGAP005661**	DNA-binding	SNP	2L-7
**AGAP005156**	DNA-binding	SNP	2L-4
**AGAP008819**	*EGFR*	SNP, Permutation	3R-6
**AGAP005147**	*FN3D1*	SNP, Permutation	2L-4
**AGAP007092**	*FN3D2*	Permutation	2L-14
**AGAP001824**	*FN3D3*	Permutation	2R-4
**AGAP011277**	*FREP6*	SNP	3L-10
**AGAP011278**	*GALE4*	SNP	3L-10
**AGAP004223**	*GPR5HT7*	SNP	2R-14
**AGAP010281**	*GPRGBB1*	SNP	3R-15
**AGAP008702**	*GPRNPR2*	SNP	3R-5
**AGAP009804**	*Gr10*	SNP	3R-11
**AGAP009805**	*Gr9*	SNP	3R-11
**AGAP005096**	Homeobox	Permutation	2L-4
**AGAP005244**	Homeodomain	SNP	2L-5
**AGAP007291**	*IAP4*	SNP	2L-15
**AGAP007292**	*IAP5*	SNP	2L-15
**AGAP007045**	*LRIM15*	SNP	2L-13
**AGAP007415**	*ML12*	SNP	2L-16
**AGAP005205**	*PGRPLA*	SNP	2L-5
**AGAP005203**	*PGRPLC*	SNP	2L-5
**AGAP012252**	*Protein C kinase 53E*	SNP	3L-19
**AGAP004375**	Ricin B lectin	Permutation	2R-15
**AGAP001004**	*TOLL1A*	SNP	X-4
**AGAP001002**	Toll-like receptor	SNP	X-4

The Gene ID is shown along with its assigned name, if any, or a homology description (Name/Description column). The SNP/Permutation analysis column indicates whether association is based on the presence of the gene within a 5 kb radius of a SNP with MAF difference >0.5 (SNP) or within a significant 10-SNP window (Permutation). The peak each gene is found corresponds to the designation shown in Figure 2.

Several putative transcription factors with homeobox-like or DNA-binding domains were found in the identified peaks. AGAP005096 in 2L-4 and AGAP005244 in 2L-5 (together with *PGRPLC* and *PGRPLA*) encode homeodomains. The homeobox gene, *Caudal*, has been previously implicated in the regulation of epithelial immune responses and shown to influence the gut bacterial population structure in *Drosophila* [28], while its mosquito homolog has been shown to regulate the IMD/REL2 pathway [49]. Thus, these putative transcription factors could play similar regulatory roles. AGAP002492 in peak 2R-7 encodes a DNA-binding domain, while its *Drosophila* ortholog *ewg* is involved in the Wnt/Wingless pathway [50]. AGAP005156, in peak 2L-4 encodes an ARID/BRIGHT DNA-binding domain, with its *Drosophila* ortholog, *retained*, is involved in behavioral modulations and repression of male courtship [51], [52]. AGAP005661, in peak 2L-7, a putative ligand-regulated transcription factor, is an ortholog of the *Drosophila* nuclear receptor *FTZ-F1*, involved in juvenile hormone mediated gene expression [53].

Genes encoding alpha-glucosidase and alpha-mannosidase homologs were detected in peaks 2R-1 and 2R-13, respectively. These genes possess glycoside hydrolase domains that are also present in the conserved chitinase gene family [54], involved in bacterial clearance and host tolerance [55].

The gene encoding the epidermal growth factor receptor, EGFR, was identified in the prominent peak 3R-6 both by both the permutation and the individual SNP analysis. The *Drosophila* EGFR pathway has been implicated in gut remodeling following oral bacterial infection [27], suggesting that the EGFR pathway may influence the outcome of *S. marcescens* infection in *Anopheles*, possibly through synergistic functions in gut homeostasis.

CLIPE6 and CLIPE7, found in peak 3L-16, belong to the non-catalytic E sub-family of CLIP-type serine proteases, a family known to participate in proteolytic cascades in antibacterial and anti-*Plasmodium* responses [56], [57], with SPCLIP1, another E sub-family member, involved in anti-*Plasmodium* responses by regulating complement recruitment [58], [59]. Several leucine-rich repeat containing genes were also detected, including *LRIM15* (peak 2L-13), a transmembrane member of the LRIM family of immune proteins [60]. LRIMs have also been implicated in complement anti-*Plasmodium* responses [61]–[63].

Two Toll-like receptors, *TOLL1A* and a previously uncharacterized paralog of *TOLL5B*, were found in peak X-4. Little is known about the role of Toll-like receptors in *Anopheles* immunity, however, cross-talk between the REL1 and REL2 signaling pathways in the yellow fever mosquito *Aedes aegypti* [64] and synergistic interactions between the Toll and Imd pathway in *Drosophila* [65], leave open the possibility for involvement of Toll-like receptors in defenses against Gram-negative bacteria, also in *Anopheles* [66].

A gene encoding a protein with a ricin B lectin domain was found in peak 2R-15. Lectins bind oligosaccharides and have been shown to modulate mosquito immune responses [6], [63], while mammalian lectins modulate host and gut microbiota interactions [67]. Genes belonging to other families of putative pattern recognition receptors were also found to be associated with the *S. marcescens* infection phenotype, including a fibrinogen-related protein (FBN or FREP) and a galectin in peak 3L-10 and an MD2-like receptor in 2L-16 [59], [68], [69].

Five annotated or putative GPCRs were found to be associated with the *S. marcescens* infection phenotype, including three putative neurotransmitter-triggered receptors: the serotonin receptor *GPR5HT7* in peak 2R-14, the GABA-B family receptor *GPRGBB1* in peak 3R-15 and the neuropeptide receptor *GPRNPR2* in 3R-5. GPCRs have been previously implicated in modulation of *P. falciparum* infection in *An. gambiae* [70], but the mechanism by which this is accomplished remains unclear. NPR-1, a neurotransmitter-triggered GPCR of *Caenorhabditis elegans*, has been shown to modulate antibacterial defenses in a behavior dependent or independent manner, and *NPR-1* genetic polymorphisms are suggested to be major determinants of bacterial susceptibility [71], [72]. Serotonin is a major modulator of mammalian intestinal inflammation [73], [74], in an interplay between the nervous and immune system [75]. The *Drosophila* ortholog of *GPR5HT7* is involved in various behavioral processes [76], [77], including aggressive behavior, a process also modulated by NPF [78]. Interestingly, the *Drosophila* ortholog of *GPRGBB1* has been implicated in behavioral responses to alcohol sensitivity [79], a process in which NPF is also a major modulator [80], [81].

Two gustatory receptor genes, *Gr9* and *Gr10*, encoding 7-transmembrane chemoreceptor domains, were associated with the outcome of *S. marcescens* infection (Figure 2, peak 3R-11). *Gr9* and *Gr10* are paralogs and show co-orthologous relationships with the *Drosophila Gr32a*, *Gr39a* and *Gr68a* [82]. Gr32a and Gr68a act as pheromone receptors in modulating mating behavior [83], [84], while Gr39a has been implicated, through 4 splice variants, in sustaining courtship behavior [85]. Gr32a is also involved in regulating aggressive behavior through recognition of small non-volatile hydrocarbons [86], or feeding suppression triggered by DEET or other antifeedants [87].

Gustatory receptor family members have also been implicated in aversive taste [35], [88], CO_2_ responses [89], [90] and sugar recognition [91]–[94]. A *Drosophila* gustatory receptor, Gr43a, has been shown to recognize fructose and act as a nutrient sensor, promoting or suppressing feeding [34]. Since enhanced or suppressed feeding of bacteria-containing sugar can decisively influence the abundance of *S. marcescens* that the mosquito takes in and its immune system can handle, it is possible that *Gr9* or *Gr10* variants linked to altered mosquito feeding behavior can affect the outcome of infection. Furthermore, GPR43, a mammalian chemoattractant receptor, has been shown to recognize short-chain fatty acids of bacterial origin and participate in antibacterial responses [36], while other mammalian chemoattractant receptors regulate inflammatory responses by recognizing endogenous factors [95]. Recognition of bacterial-derived uracil has recently been shown to modulate *Drosophila* antibacterial responses through the DUOX pathway [29]. Therefore, another possibility is that *Gr9* or *Gr10* recognize bacterial-derived metabolites or infection-induced mosquito molecules and mediate antibacterial responses.

Several other genes with no known or unrelated to immune responses homologies were also associated with the *S. marcescens* infection phenotype such as AGAP013684 in peak 2R-8, encoding a putative miRNA. MiRNAs are known to modulate gene regulation in processes that include epithelial immunity [96], [97]. AGAP006405 in peak 2L-10 encodes a tyrosine protein kinase, while its *Drosophila* ortholog, *derailed2*, is involved in Wnt5 signaling and establishment of olfactory circuits [98]. In peak 2L-15 the inhibitors of apoptosis *IAP4* and *IAP5* were found. The *Drosophila* IAP2 is known to regulate Imd signaling [99], suggesting that the *An. gambiae* IAP4 or IAP5 may also play similar roles. AGAP012252, in peak 3L-19, encodes the ortholog of *Drosophila* PKC53E, implicated in NPF-mediated alcohol sensitivity [100], [101]. AGAP011363, in peak 3L-11, encodes the ortholog of *Drosophila* rab6, implicated in phagocytosis [102] but also trafficking of Grk, the EGFR ligand [103], [104]. AGAP010503, in peak 3L-4, encodes the ortholog of the *Drosophila* SK channel, implicated in behavioral courtship memory [105]. AGAP005216, in peak 2L-5, encodes the ortholog of *Drosophila* fab1, involved in autophagy but also the lysosomal degradation of necrotic, a modulator of the Toll pathway [106]–[109].

Candidate gene prioritization for further phenotypic analysis was based on homologies with genes known to be involved in species-specific antibacterial responses, e.g. *FN3D2* and *Dscam* [43] or demonstrably regulating the response to gut microbiota in other systems, e.g. *Gr9* and the mammalian chemoattractant receptor *GPR43* [36], with the aim of the identification of novel functions of genes or gene families in antibacterial responses.

### *Serratia* infection phenotypic analysis of *FN3D1-3*

**Figure 3:**
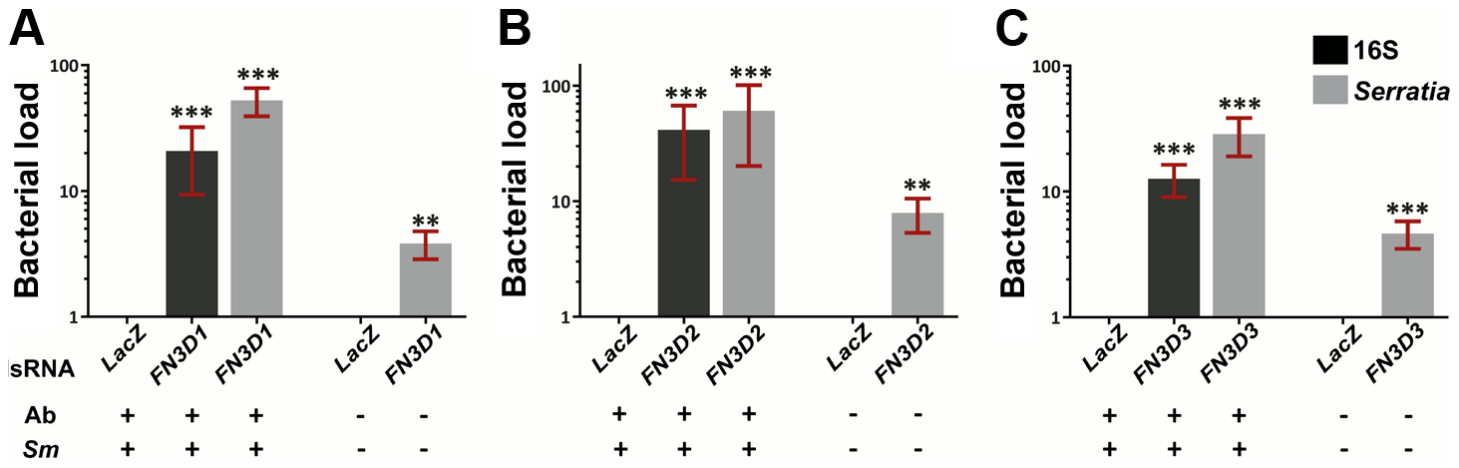
Silencing of *FN3D1–3* increases *Serratia* levels in orally infected mosquitoes or mosquitoes retaining their natural gut microbiota. Antibiotic treated and subsequently orally infected with *S. marcescens* (*Ab+Sm+*) or non-treated mosquitoes retaining their natural midgut microbiota (*Ab−Sm−*), were dsRNA treated to silence *FN3D1* (3A), *FN3D2* (3B) or *FN3D3* (3C) or treated with the *LacZ* dsRNA control. The bacterial load in the midguts of surface sterilized mosquitoes was determined 5 days post *S. marcescens* infection for *Ab+Sm+* mosquitoes, or 5 days post dsRNA treatment for *Ab−Sm−* mosquitoes. Bacterial load was determined using broad range bacterial 16S or *Serratia*-specific primers using qRT-PCR, in which relative to the endogenous *AgS7* control bacterial abundance was determined for each sample and then normalized to the relative abundance of the *dsLacZ* treated control. For *Ab+Sm+* mosquitoes, the average ±SEM of the fold-change in bacterial load is shown as determined over 7 independent infections for *FN3D1* (3A) and *FN3D2* (3B), or 8 independent infections for *FN3D3* (3C), with the qRT-PCR in each infection replicated at least twice. For *Ab−Sm−* mosquitoes, the average ±SEM of the fold-change in bacterial load is shown as determined over 4 independent assays for *FN3D1* and *FN3D3*, or 5 independent assays for *FN3D2*. Asterisks indicate significance in an one-sample t-test against zero using the log2-transformed fold-change values so that zero corresponds to no difference from *dsLacZ* treatment. Two asterisks indicate a p-value<0.005 while three asterisks indicate a p-value<0.0005.

The involvement of the three *FN3D* genes in shaping the outcome of *An. gambiae* gut infection with *S. marcescens* was investigated by RNAi-mediated gene silencing (Figure 3). Antibiotic treated mosquitoes were orally infected with *S. marcescens* following knockdown (kd) of each of the *FN3Ds* (Figure S2). The bacterial load in mosquito guts was determined 5 days post infection by quantitative RT-PCR (qRT-PCR), using both broad range bacterial 16S and *Serratia*-specific primers. Highly significant and robust increase of the *S. marcescens* load was observed after silencing any of the three genes compared to *dsLacZ*-treated controls: 21 to 53-fold in *FN3D1* (Figure 3A), 41 to 60-fold in *FN3D2* (Figure 3B) and 13 to 29-fold in *FN3D3* kd (Figure 3C).

We also assessed the role of *FN3Ds* in shaping the load of *Serratia* naturally found in the mosquito gut. Mosquitoes reared in standard conditions, without antibiotic treatment or infection with *S. marcescens*, were treated with dsRNA against each of *FN3D1–3* and the level of commensal *Serratia* was determined 5 days later (Figure 3 A–C, last bar in each panel). Silencing any of the three genes resulted in a significant 4 to 8-fold increase in the levels of commensal *Serratia* compared to *dsLacZ*-treated controls. These data indicate the involvement of FN3D1–3 in constitutive antibacterial effects that shape the load and composition of the mosquito natural gut microbiota.

### *FN3D1–3* kd alters the gut microbiota composition in favor of *Enterobacteriaceae*

When the effect of *FN3D1–3* kd was assessed on the total bacterial load in the gut of mosquitoes that retained their natural gut microbiota, a non-uniform effect was observed between 4 independent replicate assays (Figure S3). In some cases, *FN3D* silencing resulted in moderate increases of both *Serratia* and total bacterial load, while in other cases the total bacterial load showed no or marginal increase while *Serratia* showed a strong increase. This variability suggested that the FN3D effect on total bacteria may depend on the initial *Serratia* load and that FN3Ds may function in shaping the population structure of the gut microbiota by affecting a subset of bacteria inhabiting the mosquito gut, including *Serratia*.

**Figure 4:**
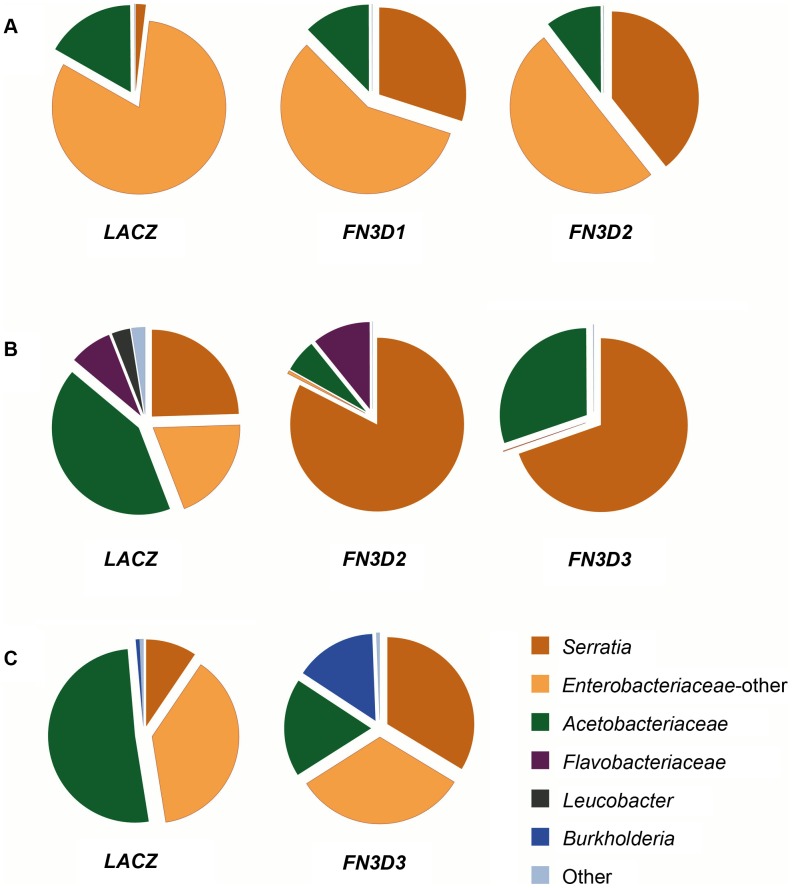
*FN3D1–3* silencing changes the composition of the mosquito gut microbiota in favor of *Enterobacteriaceae*. The 16S V4–V6 hypervariable regions of gut bacterial populations from mosquitoes retaining their natural gut microbiota without antibiotic treatment or *S. marcescens* infection (*Ab−Sm−*, Figure S3) were sequenced using 454 pyrosequencing (Table S6). cDNA pools from guts of *FN3D1–3* dsRNA treated mosquitoes or *dsLacZ* treated controls, surface sterilized and dissected 5 days post dsRNA treatment, were PCR amplified and sequenced over 3 independent assays (panels A to C). The gut microbiota composition of the *FN3D1–3* dsRNA treated pools or the *dsLacZ*-treated control in each independent assay can be seen in the respective pie charts, with the dsRNA treatment indicated below each pie chart. The color legend indicates the bacterial family corresponding to each pie chart color. Modulation of total bacteria or *Serratia* abundance can be seen for each sequenced pool in Figure S3, with *FN3D1* kd corresponding to replicate 1 in panel 4A, *FN3D2* kd corresponding to replicate 1 in panel 4A and 3 in panel 4B and *FN3D3* kd corresponding to replicate 2 in panel 4B and 4 in panel 4C.

To further investigate these hypotheses, we carried out a microbiome analysis using 454 pyrosequencing of samples from two of the replicate assays in which *FN3D1–3* kd increased *Serratia* but not total bacteria abundance (Figure 4A–B) and from a replicate assay in which *FN3D3* kd increased both *Serratia* and total bacterial load (Figure 4C). The resulting sequence reads were assigned to their respective bacterial family. Reads aligning to *Serratia* reference sequences were categorized separately from other *Enterobacteriaceae* (Table S6).

Considerable variation in bacterial composition was observed in control gut pools between the three assays (Figure 4). This variation is consistent with previously reported metagenomic analyses in lab-reared and field-collected mosquitoes, which revealed extensive gut microbiota diversity at both the individual and population levels [12]–[14]. Total *Enterobacteriaceae* (*Serratia* and other *Enterobacteriaceae*) were highly prevalent in all pools corresponding to 83.2%, 44.2% and 47.5% of total reads, respectively, while significant variation was observed in the specific representation of *Serratia* that corresponded to 1.9%, 24.5% and 9.5% of total sequence reads, respectively. This natural *Serratia* variation is consistent with the variation observed following oral infection with Db11-GFP *S. marcescens* (see Figure 1) and may be related to the underlying genetic variation. *Acetobacteriaceae* was a prominent family in all assays, while *Flavobacteriaceae* was the prevailing family in the second assay.

In the first assay, *FN3D1* or *FN3D2* kd increased the representation of total *Enterobacteriaceae* to 87.6% and 89.6%, respectively (Figure 4A). Remarkably, silencing *FN3D1* or *FN3D2* resulted in a dramatic increase in *Serratia* representation from 1.9% in the *dsLacZ*-treated control to 30% and 39.3% of total sequence reads, respectively, in agreement with the qRT-PCR analysis of the same samples (Figure S3). Similar results were obtained in the second assay whereby silencing *FN3D2* or *FN3D3* resulted in an increase in total *Enterobacteriaceae* representation, from 44.2% to 83.1% and 69.7%, respectively (Figure 4B). In both cases, *Serratia* representation showed a precipitous increase from an initial intermediate level of 24.5%, to almost all *Enterobacteriaceae* sequence reads aligning to *Serratia* reference sequences, again in consistence with the qRT-PCR analysis (Figure S3). Although non-*Enterobacteriaceae* representation decreased in both *FN3D2* and *FN3D3* kd, *Flavobacteriaceae* persisted following *FN3D2* kd but were completely eliminated following *FN3D3* kd, indicating a difference in the effect between the two *FN3D*s related to non-*Enterobacteriaceae* strains.

Taken together, these data indicate that *FN3Ds* indeed play a major role in shaping the population structure of the mosquito gut microbiota, as silencing any of *FN3D1–3* led to increased *Serratia* abundance but also shifted the composition of the mosquito gut microbiota in favor of *Enterobacteriaceae*, mainly *Serratia* or strains that show similarity to *Serratia* reference sequences. This shift may be a result of a specific FN3D function against *Serratia* or a subset of gut bacteria. Alternatively, bacterial interactions or differential growth potential of different bacterial strains may account for the observed shift following a uniform FN3D antibacterial effect.

We tested this hypothesis by examining whether *FN3D1–3* silencing could affect the levels of gut infection with non-*Enterobacteriaceae*. Antibiotic treated *dsLacZ* treated controls and *FN3D1–3* kd *An. gambiae* mosquitoes were orally infected with bacteria of the genus *Asaia*, a member of the *Acetobacteriaceae* family, common in both field and laboratory-reared *An. gambiae* [9]–[11] and present in all of our sequenced samples. *FN3D1–3* silencing resulted in moderate, non-significant increases in bacterial load, compared to controls (Figure S4), distinguishably lower than following oral *S. marcescens* infection (Figure 3). These data suggest that the observed FN3D antibacterial effect is not uniform across all Gram-negative bacteria and may be specific to a subset of the gut bacterial population including *Enterobacteriaceae*.

The observed shift in favor of *Enterobacteriaceae* representation when both *Serratia* and total bacterial abundance increased following *FN3D3* kd was also confirmed by microbiome sequencing that showed an increase of *Serratia* from 9.5% to 33.7% of total sequence reads and of total *Enterobacteriaceae* from 47.5% to 66% (Figure 4C). Remarkably, *FN3D3* kd also increased the representation of bacteria of the genus *Burkholderia*, from an initial 0.71% to 15.1% of total reads (Figure 4C). *Burkholderia* were not traced in the *dsLacZ* treated control pool in which the effect of *FN3D3* kd was also assayed (Figure 4B). These data suggest that FN3D3 limits a subset of the mosquito gut bacterial community including *Enterobacteriaceae* but also bacteria of the genus *Burkholderia*.

### Gr9 modulates *S. marcescens* infection levels

The genomic area encompassing genes encoding the gustatory receptors Gr9 and Gr10 was associated with the outcome of *S. marcescens* infection. As alternative splicing of *Gr9* has been previously suggested [82], with *Gr9* possessing 13 splice variants compared to one for the adjacent *Gr10*, we considered *Gr9* genetic variation more likely to influence the outcome of *S. marcescens* infection, leading to the observed SNP divergence. *Gr9* has shown significant upregulation compared to other tissues in the midgut of blood-fed adult mosquitoes [110] and also in the midgut of adult mosquito tissues [111]. The *Gr9* midgut expression was also confirmed here (Figure S2). Furthermore, comparison of transcription profiles between antennae or maxillary palps and whole body transcriptomes in female mosquitoes has previously shown a non-significant upregulation of *Gr9* in those two tissues (1.42 for antennae and 1.15 for maxillary palps) [112].

**Figure 5:**
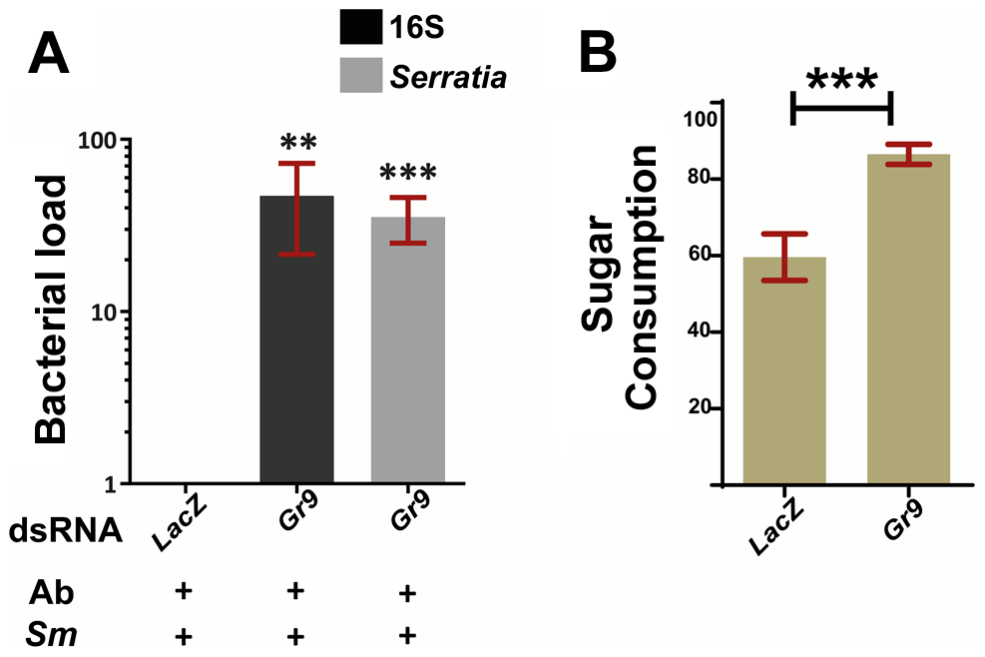
*Gr9* silencing increases *S. marcescens* levels in orally infected mosquitoes. **5A:** The bacterial load of antibiotic treated mosquitoes orally infected with *S. marcescens* (*Ab+Sm+*), treated either with *Gr9* dsRNA or the *dsLacZ* control was determined at day 5 post infection either with broad range 16S or *Serratia*-specific primers. The average ±SEM of the bacterial fold-increase is shown, compared to *dsLacZ* treated mosquitoes over 5 independent infections, with the qRT-PCR performed at least twice for each infection. **5B:** Antibiotic treated mosquitoes were starved overnight and then offered a sugar meal through a 5 µl capillary. Sugar meal consumption was determined 16 hours later for 38 *LacZ* and 55 *Gr9* dsRNA treated mosquitoes. The average ±SEM percentage of sugar consumption through the capillary for each mosquito is shown for *LacZ* and *Gr9* dsRNA treatments. In panel 5A, asterisks indicate significance in a one-sample t-test against zero using the log2-transformed fold-change values while in panel 5B asterisks indicate significance in the non-parametric Mann-Whitney test. Two asterisks indicate a p-value<0.005 while three asterisks indicate a p-value<0.0005.

We carried out RNAi-mediated silencing of *Gr9* in adult mosquitoes and examined the outcome of oral *S. marcescens* Db11-GFP infection. *Gr9* knockdown resulted in a precipitous 36 to 48-fold increase in *S. marcescens* levels compared to *dsLacZ* treated controls, as determined using both broad range 16S and *Serratia*-specific primers (Figure 5A). These data suggest that Gr9 exerts an antibacterial effect that influences the outcome of *S. marcescens* infection.

We next examined the possibility that the Gr9 antibacterial effect is related to changes in mosquito feeding behavior. Based on the *Gr9* many-to-many orthologous relationship with the *Drosophila Gr32a*, *Gr39a* and *Gr68a*, it would be more likely that any Gr9 effects on mosquito behavior would be exerted through the recognition of mosquito-induced or bacterial-derived molecules rather than nutrient sensing [83]–[85], [87].Therefore we first examined the possibility that Gr9 mediates aversion to bacteria-containing sugar thus limiting sugar meal uptake upon oral *S. marcescens* infection. A two-choice preference assay, in which mosquitoes were offered to feed from a capillary that contained *S. marcescens* and another that contained only sugar, indicated that there was no significant difference due to *Gr9* silencing in consumption between the two capillaries (Figure S5).

Another possibility that could explain the Gr9 antibacterial effect is that Gr9 modulates meal size irrespective of the presence of bacteria. Antibiotic treated mosquitoes were starved and then offered a sugar meal. Consumption was determined 16 hours later in *LacZ* or *Gr9* dsRNA treated mosquitoes (Figure 5B). Indeed, *Gr9* silencing resulted in a significant 1.45-fold increase in meal size compared to *dsLacZ* treated mosquitoes. An increased meal size could result in higher *S. marcescens* uptake following oral infection, thus contributing to the precipitous increase in *S. marcescens* load following *Gr9* silencing. Our data suggest that Gr9 influences feeding behavior by triggers that do not rely on the presence of bacteria. As the presence of *S. marcescens* does not seem to affect sugar uptake following *Gr9* silencing, there is no reason to assume that the presence of *S. marcescens* influences the Gr9 effect on meal size, although Gr9-independent aversion circuits could conceivably taper overall consumption.

### Transcriptional responses following *S. marcescens* infection

**Figure 6:**
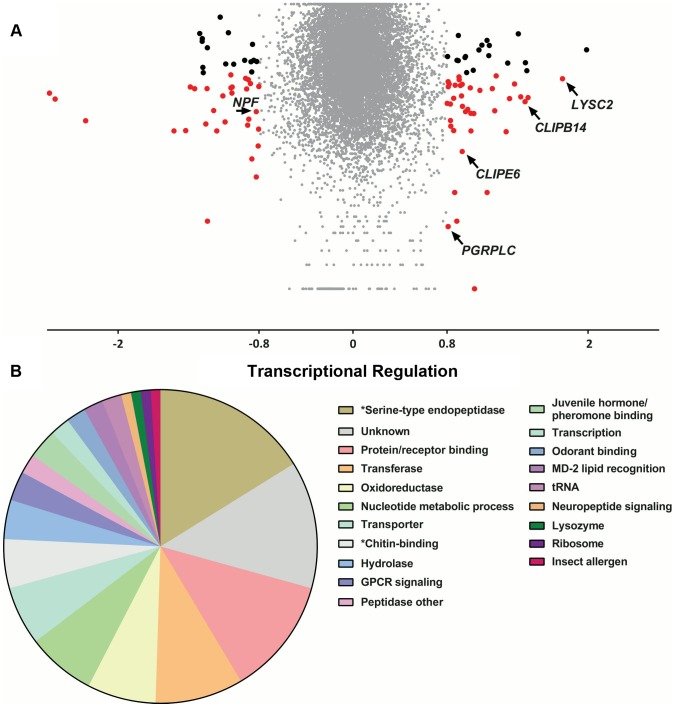
Transcriptional regulation following *S. marcescens* infection using DNA microarrays. Antibiotic treated mosquitoes were orally infected with *S. marcescens* and, 3 days post infection, transcriptional regulation in the gut of bacteria-fed mosquitoes was determined using DNA microarrays, compared to uninfected mosquitoes further antibiotic treated for 3 days. **6A:** Volcano plot of transcriptional regulation as determined over 3 independent infections. The log2-transformed fold-change values for each transcript, as determined by two probes for each of the three arrays, were used for a one-sample t-test against zero, where zero corresponds to no regulation. Transcripts with more than 1.75-fold regulation are indicated either by black dots if the p-value of the t-test is >0.05 or red dots if the p-value is <0.05. Transcripts corresponding to *LYSC2*, *PGRPLC*, *CLIPE6*, *CLIPB14* and *NPF* are indicated by arrows. **6B**: Functional classification of more than 1.75-fold regulated genes. The 97 genes with more than 1.75-fold regulation were assigned to a functional class based on assigned GO terms, *InterPro*-predicted domains or *Drosophila* orthologs. The pie chart shows the proportion of genes assigned to each functional class. Functional classes corresponding to significantly overrepresented GO terms are indicated by asterisks.

To examine the relationship between genes identified in the population genetics analysis to be associated with the *S. marcescens* infection outcome and infection-induced transcriptional responses, we used DNA microarrays to monitor the transcriptional profile of mosquito guts 3 days post infection with *S. marcescens* added to the sugar meal. Uninfected mosquitoes, which were also treated with antibiotics, were used as controls. Three independent replicate infections were performed. Overall, 55 and 44 transcripts were found to be up and down regulated by at least 1.75-fold, respectively, with 38 and 28 respective up or down regulated transcripts yielding a significant p-value in a t-test against zero, where zero corresponds to no transcriptional regulation (Figure 6A and Table S7). Functional classification of all 97 differentially regulated genes, accounting for multiple transcripts of the same gene, identified serine-type endopeptidases and protein/receptor binding as the most represented classes (Figure 6B). The protein/receptor binding functional class comprised 12 members, including several up or downregulated FREPs, zinc finger containing proteins, PGRPLC and the complement factor regulator LRIM1, which has been previously shown to be regulated by the IMD/REL2 pathway [113]. The oxidoreductase class comprised 7 members, including two P450 cytochromes, possibly involved in detoxification [114], the hydrolase class included a glycoside hydrolase and the nucleotide metabolic process class included 5 heat shock proteins, likely to be involved in stress responses [115]. The antimicrobial peptide *LYSC2*, showing the highest 3.44-fold upregulation of all genes, has been previously shown to be upregulated following a bacterial challenge [116].

A hypergeometric test followed by Benjamini-Hochberg correction was used to determine enriched GO terms in the set of 97 genes. The results identified 16 GO terms that were significantly overrepresented, most of which were related to just two functional classes: serine-type endopeptidases and chitin-binding genes (Figure S6 and Table S8). In total, 16 serine-type endopeptidase genes were differentially regulated, including *CLIPE6*, which was also associated with the outcome of infection, *CLIPB14* that has been implicated in defense against Gram-negative bacteria [57], [117], *CLIPB17* and *CLIPB20*. The group of chitin-binding genes comprised 5 members, including the gene encoding the scavenger receptor SCRASP1, previously shown to be upregulated following bacterial infection and bind chitin [117], [118] and two downregulated peritrophic matrix components identified by a previous proteomic analysis [119]. Chitin-binding genes are upregulated following oral bacterial infection in *Drosophila* [26], with one member participating in barrier formation that protects against oral *S. marcescens* infection [120], while their suggested role in mosquitoes is recognition of danger signals following tissue remodeling due to a bacterial infection [118].

Several transcriptionally regulated genes suggested a mosquito behavioral response following *S. marcescens* infection (Table 2). Among these genes, *NPF* was downregulated after infection. *NPF* is expressed in the midgut of *Drosophila* [121] and *Aedes aegypti* [122], and has been implicated in modulation of feeding behavior in *Drosophila* [30], aversion to noxious food [123] as well as in alcohol sensitivity [80] and regulation of reward systems [81]. It has been also linked to food signaling by integrating sugar gustatory stimuli [124] and behavioral immune responses against endoparasitoid wasps, by mediating oviposition behavior [125].

**Table 2:** Transcripts related to mosquito behavior regulated more than 1.75-fold following *S. marcescens* oral infection.

Transcript ID	Name/Description	Functional class	Fold-change	Regulation
**AGAP004642-RA**	*NPF*	Neuropeptide signaling	1.77	down
**AGAP002635-RB**	*Gr13*	GPCR signaling	2.68	down
**AGAP002635-RA**	*Gr13*	GPCR signaling	2.13	down
**AGAP008052-RA**	Insect pheromone-binding protein	Juvenile hormone/pheromone binding	1.77	down
**AGAP008182-RA**	Juvenile hormone-binding	Juvenile hormone/pheromone binding	2.06	down
**AGAP006080-RA**	*OBP54*	Odorant binding	2.38	down
**AGAP002905-RA**	*OBP13*	Odorant binding	6.00	down
**AGAP012703-RA**	*TO2*	Juvenile hormone/pheromone binding	1.78	up
**AGAP003759-RA**	CHK kinase-like juvenile hormone-inducible	Transferase	1.81	up
**AGAP003220-RA**	CHK kinase-like juvenile hormone-inducible	Transferase	1.94	up
**AGAP003762-RA**	CHK kinase-like juvenile hormone-inducible	Transferase	2.04	up

The Transcript ID is shown along with its assigned name or homology description, the assigned functional class and the fold-change transcriptional regulation as determined over three independent infections.

Additional behavior-related genes that were transcriptionally regulated following *S. marcescens* infection included the gustatory receptor *Gr13* with two downregulated transcripts, three upregulated juvenile hormone-inducible kinases, two downregulated genes encoding a pheromone and a juvenile hormone binding protein and the downregulated odorant binding protein genes *OBP13* and *OBP54*. Juvenile hormone circuits are known to affect gustatory perception and feeding behavior in various organisms including *Ae. aegypti* [126]–[129], while pheromone and olfaction circuits are also known to affect mosquito behavior [130], [131]. The gene encoding the juvenile hormone binding protein TO2 (takeout2), was also upregulated following *S. marcescens* infection and may also participate in behavioral responses, as its *Drosophila* homolog, takeout, is known to regulate feeding behavior [132], [133].

### Gr9 modulates *S. marcescens* infection via NPF

**Figure 7:**
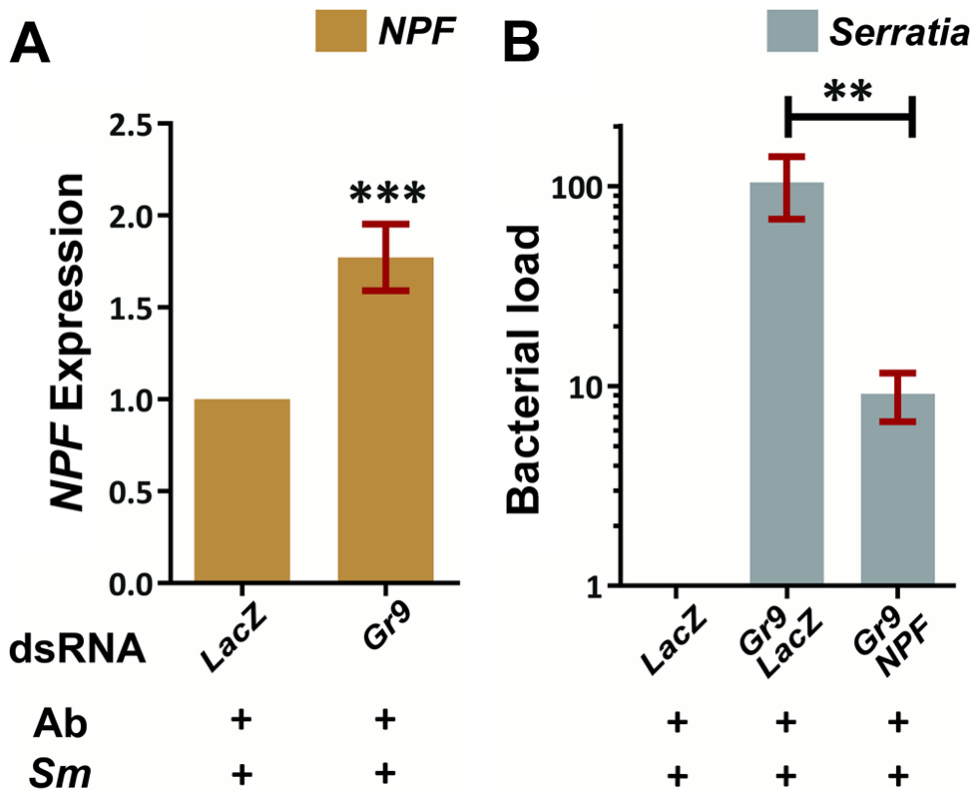
The Gr9 antibacterial effect mostly relies on changes in *NPF* expression. **7A:** Antibiotic treated mosquitoes orally infected with *S. marcescens* (*Ab+Sm+*) were dissected 5 days post infection and the *NPF* levels of mosquitoes treated either with *LacZ* or *Gr9* dsRNA were determined in their guts. The average ±SEM of the *NPF* fold-increase as determined over 8 independent infections, with the qRT-PCR performed at least twice for each infection, can be seen. **7B**: The bacterial load of antibiotic treated mosquitoes orally infected with *S. marcescens* (*Ab+Sm+*) treated either with *LacZ* dsRNA or with a 50–50% mix of either *Gr9* and *LacZ* or *Gr9* and *NPF* dsRNA was determined and normalized to the levels of *dsLacZ* treated mosquitoes. The average ±SEM of the bacterial fold-increase as determined over 3 independent infections can be seen, with the qRT-PCR performed at least twice for each infection. In panel 7A asterisks indicate significance in a one-sample t-test against zero using the log2-transformed fold-change values while in panel 7B asterisks indicate significance in the non-parametric Mann-Whitney test. Two asterisks indicate a p-value<0.005 while three asterisks indicate a p-value<0.0005.

We examined a possible link between the observed downregulation of the known modulator of feeding behavior, *NPF*, following *S. marcescens* infection and the role of Gr9 in modulating the *S. marcescens* infection outcome. The *Gr9* ortholog *Gr39a* has been previously shown to be expressed in the *Drosophila* midgut and co-localize with NPF in enteroendocrine cells [134], raising the possibility of a functional link between these two genes. In mosquitoes orally infected with *S. marcescens*, *NPF* expression showed a significant 1.8-fold increase following *Gr9* silencing compared to *dsLacZ* treated controls (Figure 7A). This modulation of *NPF* expression following *Gr9* silencing suggested that *NPF* expression may mediate the observed *Gr9* antibacterial effect. Therefore, we further examined whether silencing *NPF* can affect the increase of *S. marcescens* observed in *Gr9* kd mosquitoes. Indeed, concomitant silencing of *Gr9* and *NPF* resulted in a significant 10-fold decrease in infection load, compared to *Gr9* silencing alone (Figure 7B).

Taken together, our data suggest a behavioral immune response involving Gr9, mostly relying on changes of *NPF* expression. One hypothesis is that Gr9 activation in the midgut, most likely through a mosquito-induced cue, tapers the expression of *NPF*, resulting in feeding suppression that limits the mosquito meal size and thus the abundance of ingested *S. marcescens*. *Gr9* variants that influence the efficiency of this suppression may lead to enhanced feeding which, depending on the efficiency of the epithelial response to handle the infection, can influence the outcome of *S. marcescens* infection, thus explaining the observed *Gr9* association with the *S. marcescens* infection phenotype.

## Discussion

The rapidly evolving and adapting mosquito species have become tractable systems for genetic association studies that could yield important information about vector/parasite interactions leading to malaria transmission [135]. Previous studies have focused on the outcome of *Plasmodium* infections, using laboratory or field mosquitoes and genetic tools such as microsatellite markers and targeted SNP loci genotyping [1], [3], [136]. These studies have not considered the effect of gut bacteria on the outcome of *Plasmodium* infections, which has been revealed recently [5], [16]–[18]. Furthermore, the influence of associated complement factors on natural *P. falciparum* infections remains questionable [137]. Indeed, the presence of *Enterobacteriaceae*, such as *S. marcescens*, a common member of the mosquito gut flora, has been correlated with *P. falciparum* susceptibility in field mosquito populations [12], while intraspecific variation within *S. marcescens* populations also is shown to affect the *Plasmodium* infection load [37]. Therefore, genome-wide studies to determine factors that modulate the levels of mosquito gut bacteria can provide novel insights into how midgut bacteria affect the outcome of *Plasmodium* infection and hence malaria transmission.

The unprecedented level of detail achieved in the population genetics analysis presented here in identifying SNPs associated with the outcome of *S. marcescens* infection is a result of the strong evolutionary drive exerted by gut bacteria on mosquito genetic variation, the use of a high-resolution SNP genotyping array and the use of a recently established laboratory colony of *An. gambiae* which retains genetic variation found in field populations but also shows elevated linkage disequilibrium due to colonization bottlenecks. This population homogeneity can facilitate gene discovery as shown in human genome-wide association studies in isolated populations [138], [139].

A dual implication can be inferred for genes associated with the *S. marcescens* infection phenotype; they are putatively involved in shaping the infection outcome, while their level of involvement may also be affected by genetic variation within the mosquito population. It is possible that identified associations are the result of causal polymorphisms such as gain or loss of function mutations in coding or regulatory sequences or the result of allele combination in several genetic loci which shapes the outcome of infection through synergism, epistatic interactions or redundant function. In any of the latter cases, a reverse genetics approach may not be capable of capturing such interactions.

The involvement of the three *FN3D*s in the outcome of *Serratia* infection reveals a novel function of this family in modulating the load and composition of the mosquito gut microbiota and opens new avenues in investigating the complexity of such responses and possible synergisms with known antibacterial pathways such as the IMD/REL2. The three *FN3D* genes identified here emerge as major modulators of the bacterial population structure in the mosquito gut, limiting the representation of *Enterobacteriaceae*, mainly *Serratia* or strains with similarity to *Serratia* reference sequences, but also, for *FN3D3*, bacteria of the genus *Burkholderia*. As shifts in gut microbiota population structure can elicit gut pathology [28], [140], while *Serratia* can influence the outcome of *Plasmodium* infection [37], FN3Ds can play critical roles in gut homeostatic interactions and malaria transmission dynamics.

Further insights into the FN3D mode of action remain to be determined. Our data showing that the knockdown effects of *FN3Ds* may be limited to *Serratia* or to a fraction of the microbiota raise intriguing questions about the specificity of bacterial recognition in the mosquito gut. The homology of *FN3D2* with the hypervariable pattern recognition receptor *Dscam* opens the possibility that the specific pathogen recognition shown for *AgDscam* [47] concerns a broader family of *FN3Ds*, equipping mosquitoes with the capacity for specific recognition resembling that of animals possessing adaptive immune systems. The phylogenetically unrelated *FN3D2* and *FN3D3* share a similar domain architecture comprising immunoglobulin and FN3 domains, as is the case with *Dscam*. The identification of *FN3D2* and *FN3D3* as being both associated with the outcome of *S. marcescens* infection and exhibiting discrete but similar phenotypic characteristics in modulating the bacterial population structure in the mosquito gut, parallels the discrete but similar functions of the phylogenetically unrelated *Dscam*, *Frazzled* and *Roundabout* in *Drosophila* axon guidance, with all three receptors sharing immunoglobulin and FN3 domains [141]–[144]. *FN3D1* has a distinct domain architecture with an FN3 domain, while its orthologous relationship with *Drosophila windei* [145] and sequence similarity with the activating transcription factor 7- interacting protein [146], suggest a role in regulating gene expression.

The identification of *An. gambiae* genes involved in immune responses against bacteria and/or *Plasmodium* has been largely based to date on studies that combine bioinformatic identification of known immunity gene homologs and transcriptional profiling of genes following a pathogen challenge. This approach, however, has the limitation of the *a priori* assumption that genes of interest show significant change in transcriptional regulation, mostly induction, which is true for most effectors, but not all genes, for example pattern recognition receptors or transcription factors. In addition, it is possible that even strong changes in transcriptional regulation are the consequence of the infection rather than part of the response. Especially for quantitative traits within mosquito populations, such as *Plasmodium* infection intensity, different infection intensities can correlate with variable transcriptional responses [70], while the underlying genetic variation further complicates the observed transcriptional regulation.

The microarray approach adopted here has identified a limited set of 99 differentially regulated transcripts following oral *S. marcescens* infection. The number of regulated transcripts is consistent with that of a previous microarray-based comparison of antibiotic treated and untreated mosquitoes, which showed differential expression for 185 transcripts [16], attributing this limited transcriptional regulation to symbiotic relationships that have led to adaptation of commensal bacteria. A much broader set of differentially expressed genes has been identified following oral bacterial infections in *Drosophila* [26], [32]. This is most likely due to differences in gene pool diversity between the genetically homogeneous fly lines and the recently established mosquito laboratory colony used here, which retains considerable genetic variation thus enabling the SNP genotyping analysis. The different levels of infection seen between mosquitoes (high, low and no infection), which are largely attributed to genetic variation within the colony population, are most likely linked to differences in the mosquito transcription profiles that are averaged out in our study design. Therefore, our analysis identifies transcripts with the most pronounced and consistent differential expression, comprising the core response to *S. marcescens* infection. Future studies investigating the transcription profile of highly, lowly or non-infected mosquitoes are most likely to reveal components of transcriptional regulation that lead to the respective outcome of infection. Indeed, genes identified to show prominent differential expression after bacterial challenge in previous studies also showed transcriptional regulation following oral *S. marcescens* infection, including *CLIPB14* [117], *LRIM1* [63], [113], *LYSC2* [116] and *SCRASP1* [117], [118].

The identification of diverse transcriptional responses to different bacteria in *Drosophila* [32] along with the specificity of mosquito responses to a subset of bacteria, as suggested by the SNP genotyping analysis presented here, may explain the surprisingly little overlap between differentially expressed genes following *S. marcescens* infection and antibiotic treated vs. untreated mosquitoes [16]. Remarkably, however, consistency is seen in gene families present in both datasets, including CLIPs, chitin-binding genes, homeobox genes, *PGRPs* and *FREPs*, suggesting that similar defense strategies are employed, which are customized for each type of infection through utilization of different gene family members.

The approach we adopted here to identify genes involved in mosquito gut infection with *S. marcescens* combines transcriptional profiling of infected guts with the identification of SNPs segregating between phenotypic pools, whereby an association implies contribution to the outcome of infection, while the study design incorporates variation that leads to different observed phenotypes. This approach addresses some of the aforementioned shortcomings but introduces others, as it cannot capture genes with redundant functions, genes with additional housekeeping functions or a role during development, of which variants are eliminated from the population, or genes with rare variants that are not in the variation pool of our colony. Furthermore, an association may be the result of a selective sweep in the proximity of the gene that creates linkage disequilibrium and leads to SNP divergence between the phenotypic pools. Therefore, although each of the approaches cannot provide by itself a complete picture, the combination of the two can provide novel insights into the mosquito gut responses to *S. marcescens*.

The comparison between the datasets of transcriptionally regulated genes and genes associated with the outcome of *S. marcescens* infection shows limited overlap, with only *PGRPLC* and *CLIPE6* found in both datasets. Again, considerable overlap is detected in identified gene families, which are represented by different members in each dataset. These include acyl-transferase, glycoside hydrolase, kinase, GPCR, LRIM, homeobox, zinc-finger, PGRP, peptidase, FREP, MD2-like and chitin-binding genes. Interestingly, a previous study investigating differential expression following a bacterial challenge in mosquito immunoglobulin-containing genes failed to identify significant regulation for *FN3D2* or *FN3D3* [147], strengthening the case for the complementarity of the SNP genotyping and expression analysis approaches. The specific role of gene family members, especially those showing considerable expansion in *Anopheles*, e.g. FREPs [68], [148], remains unclear. Therefore, SNP genotyping reveals a different set of candidate genes involved in antibacterial immunity while at the same time it is intriguing to postulate whether this divergence between associated and differentially expressed genes within each gene family constitutes a functional divergence between them.

A novel finding stemming from this combinatorial approach is a mosquito behavioral response to *S. marcescens* infection that involves *Gr9* signaling and is mediated by changes of *NPF* expression. Although *Gr9* orthologs in *Drosophila* recognize chemosensory cues and mediate aversive behaviors [83], [84], [87], surprisingly, Gr9 appears to suppress feeding irrespective of the presence of bacteria. One explanation is that the Gr9 antibacterial effect relies on its expression in the midgut rather than external sensory organs, where the role of its *Drosophila* counterparts has been studied. The role of gustatory receptor midgut expression [134] remains poorly understood and could involve detection of nutrients or host-derived molecules that triggers downstream responses. The role of *NPF* midgut expression [121], [122] also remains poorly understood. *NPF* downregulation following *S. marcescens* infection implies its involvement in an aversion circuit triggered by the presence of *S. marcescens*, with a possible NPF role in integrating aversion and satiation signals that lead to feeding suppression. Such NPF involvement remains to be further investigated, in conjunction with the involvement of other genes related to mosquito behavior which were either associated with the outcome of *S. marcescens* infection or were transcriptionally regulated following infection. These include *Gr13*, downregulated following *S. marcescens* infection but also three neurotransmitter-triggered GPCRs, associated with the outcome of infection, pointing to complex behavioral circuits involved in antibacterial responses, which are yet to be revealed.

The identification of *FN3Ds* as well as *Gr9* and *NPF* in responses affecting the outcome of *S. marcescens* infection, in addition to known responses including the IMD/REL2 and DUOX pathways, suggests that the response to gut infection is the result of a complex molecular interplay. Both the SNP genotyping and expression analysis suggest that the mosquito response to oral *S. marcescens* infection involves two discrete but inextricably linked modes of defense, a behavioral and an epithelial immune response. A behavioral immune response involving Gr9 and NPF can limit or disrupt pathogen intake, a defense conceptually similar to barrier responses that inhibit pathogen contact with triggers of epithelial or systemic immune responses. An impaired behavioral response, e.g. due to *Gr9* variants that affect feeding behavior, can decisively influence the efficacy of the epithelial response and thus the infection outcome. This implies a threshold after which epithelial immunity cannot efficiently handle the pathogen load, an aspect of immunity that remains poorly understood. Nevertheless, in mosquito infections with *Plasmodium* parasites, the intensity of infection has been correlated with the efficacy of different components of the IMD/REL2 pathway, suggesting that different effectors may be deployed in low, mid or high intensity parasite infections [4].

As pathogen abundance most likely relies on feeding behavior, the interplay between behavioral and epithelial immunity can shape both responses. Our implementation of a model of natural bacterial infections through the oral route integrated both behavioral and epithelial responses and not only revealed the previously unknown behavioral component but also allowed the study of aspects of epithelial immunity that, by being infection intensity dependent, possibly rely on the behavioral component. This integrative approach to behavioral and epithelial immunity can be further employed to reveal aspects of this interplay that may involve regulation of behavioral responses by host-derived factors induced by the epithelial component. This implies that the study of behavioral immunity alone may be insufficient to uncover some aspects of its biological consequences.

In *Drosophila*, a balance between immune response and tolerance, achieved by various Imd regulators, largely shapes the gut microbiota population structure, although the only known elicitor of such responses is DAP-type peptidoglycan, common to all Gram-negative bacteria [28], [149], [150]. A similar mechanism has been suggested for mosquitoes through alternative splicing of the modular IMD/REL2 pathway receptor *PGRPLC* that leads to production of positive and negative pathway modulators [5], [42]. Indeed, utilization of alternative splicing as a mechanism to derive new immune functions and increase the specificity of pathogen recognition by the mosquito innate immune system has been described for the *FN3D2* homolog, *Dscam* [43], [44], [47]. Whether the *Enterobacteriaceae*-specific effect of *FN3D2* knockdown is due to specific recognition and activation of highly specialized or targeted effector reactions remains to be investigated. Furthermore, the significance of alternative splicing suggested for *Gr9* [82] remains to be determined along with the cue that triggers its antibacterial effect, and could also involve recognition of, most likely, host-derived signals. In addition, recognition of differentially produced metabolites after infection as shown for the DUOX pathway [29] could further increase the specificity in antibacterial responses. Whether PAMPs (bacterial-derived) or DAMPs (host-derived), such metabolites can be recognized by gustatory receptors triggering specific antibacterial responses, which together with *FN3Ds* and the rather generalist response of the IMD/REL2 pathway can shape the load and composition of the mosquito gut microbiota. In conclusion, our findings suggest that mosquitoes can mount a much more complex and specific antibacterial response than previously thought, which not only contributes to fending off intestinal bacterial infections but also to achieving homeostasis of the complex gut ecosystem.

## Materials and Methods

### Ethics statement

This study was carried out in strict accordance with the United Kingdom Animals (Scientific Procedures) Act 1986. The protocols for maintenance of mosquitoes by blood feeding were approved and carried out under the UK Home Office License PPL70/7170. The procedures are of mild to moderate severity and the numbers of animals used are minimized by incorporation of the most economical protocols. Opportunities for reduction, refinement and replacement of animal experiments are constantly monitored and new protocols are implemented following approval by the Imperial College Ethical Review Committee.

### Mosquito rearing and maintenance

The *N'gousso* strain of *An. gambiae* was used. This is an M form strain colonized in 2006 [1] and kept in large numbers to retain genetic variation. Rearing and maintenance of the strain was performed as described previously [151]. Mosquitoes were collected after emergence and kept on a cocktail of 25 µg/ml gentamicin, 10 µg/ml penicillin and 10 units/ml streptomycin, diluted in 10% D-(-)-Fructose (Sigma). This antibiotic treatment regime was used for 5 days, with the antibiotic solution refreshed every 24 hours. At day 5 post emergence, the antibiotic solution was replaced by dH_2_O and mosquitoes were starved overnight prior to oral bacterial infection.

### Mosquito oral infection with *S. marcescens* or *Asaia*

We used the *S. marcescens* Db11-GFP strain, modified to be GFP-fluorescent and resistant to tetracycline and carbenicillin [152]. *S. marcescens* glycerol stock was grown in 5 ml LB cultures containing 50 µg/ml tetracycline and carbenicillin (Sigma) at 37°C. Following overnight incubation, the cultures were expanded to 100 ml and further incubated overnight at 37°C. OD_600_ and GFP fluorescence (excitation/emission at 485/520 nm) were then measured to ensure cultures maintained GFP fluorescence, using the Fluostar Omega spectrophotometer (BMG Labtech). Bacterial pellets following centrifugation at 2500 rpm for 5 minutes were washed twice with PBS and resuspended in such volume of 10% D-(-)-Fructose, so that 1 ml of the bacteria-containing sugar solution corresponded to OD_600_ = 0.1 of the initial 100 ml culture. The sugar solution was further diluted 1∶12 in a 10% D-(-)-Fructose solution that contained tetracycline and carbenicillin at 50 µg/ml and 5% v/v of scarlet dye (Langdale). Mosquitoes were fed with this solution for 2 days. Subsequently, mosquitoes fed with bacteria-containing sugar were separated based on the presence of the dye in their gut and kept on 10% D-(-)-Fructose containing tetracycline and carbenicillin at 50 µg/ml.

Oral infections with *Asaia* were conducted in a similar manner. The *Asaia* SF2.1 (GFP) strain was used, grown as previously described [153] and maintained in 50 µg/ml kanamycin (Sigma).

### Fluorescence microscopy

The levels of *S. marcescens* infection were determined by microscopic observation of dissected midguts immersed in *Vectashield* mounting medium (Vecta), immediately after dissection. The Zeiss Axiophot fluorescence microscope was used, equipped with light and GFP filters while photos of observed midguts were taken with the Axiocam HRc and Axiovision software (Zeiss).

### SNP genotyping arrays

All carcasses corresponding to midguts of *S. marcescens* infected mosquitoes were kept numbered in 96-well plates immersed in 75% ethanol at −80°C. Carcasses from selected midguts were used for gDNA extraction using the *QIAquick Blood and Tissue* kit (QIAGEN). Subsequently, gDNA concentrations were determined using the *Picogreen dsDNA* kit (Invitrogen) and equimolar gDNA quantities from each mosquito were pooled. The design and validation of the SNP genotyping array used along with the treatment of gDNA pools, hybridization, calling of SNP genotypes and measurement of differentiation in each pooled hybridization between allele A and B have been described previously [39], [154]. The frequency of designated allele A was considered as the minor allele frequency and was used to measure the difference between pooled hybridizations. The permutation analysis used has been described previously [39], with a modified length of non-overlapping 10-SNP windows. Determination of genes residing in identified genomic areas and homology analysis was performed using Biomart 0.7 and the AgamP3.7 *An. gambiae* gene annotation [155]. The SNP genotyping array datasets have been deposited to ArrayExpress under the experiment name *Serratia_SNP1* and accession number E-MEXP-3951.

### DNA microarrays

Total RNA was extracted from midguts using the *Trizol* reagent (Invitrogen), and treated with *Turbo DNAse I* (Ambion). Samples were further purified using the *RNeasy* kit (QIAGEN). Quantification was performed using the *Nanodrop 1000* spectrophotometer (Thermo Scientific) and RNA integrity was assessed using the *RNA 6000 Pico Chip* kit (Agilent). Labeling and hybridization were performed using the *Low Input Quick Amp Labeling* kit for two-color microarray based expression analysis (Agilent). We used Agilent custom 4×44 k gene expression microarrays. The microarray design *Pfalcip_Agamb200*9 (A-MEXP-2324) comprises oligonucleotide probes encompassing all *An. gambiae* annotated transcripts of the AgamP3.6 release along with *P. falciparum* probes, with each probe represented in duplicate. Slides were scanned using the *Genepix 4000B* scanner equipped with the *Genepix Pro 6.1* software (Axon instruments).

All dataset files were normalized using the *Genespring 11.0 GX* software (Agilent). The Lowess normalization method was used while the threshold of raw signals was set to 5, which was sufficient to eliminate background regulation of *P. falciparum* probes. Further analysis of transcriptionally regulated genes and GO analysis was performed using the Genespring 11.0 GX software. For GO analysis, GO accession numbers for all *An. gambiae* transcripts were obtained using Biomart 0.7 and a hypergeometric test with Benjamini-Hochberg correction was performed on the set of more than 1.75-fold regulated genes. The corrected p-value for testing multiple GO accession numbers for their significance was set to 0.1. The log2-transformed transcriptional regulation for each transcript was extracted from the normalized datasets for each of the two probes corresponding to each transcript and the obtained values from all three independent infections were used in a t-test against zero, with a p-value cut-off of 0.05. The DNA microarray datasets have been deposited to ArrayExpress under the experiment name *Serratia_infections* and accession number E-MEXP-3952.

### Bacterial load following RNAi-mediated silencing

Mosquitoes were treated with the respective dsRNA at the day of emergence, as described previously [156]. For each targeted gene or the *dsLacZ* control, dsRNA was synthesized using the *T7 Megascript* kit (Invitrogen) and further purified using the *RNeasy* kit (QIAGEN) to a concentration of 3 µg/µl. For each *T7 Megascript* reaction, 1 µg of purified PCR product was added, derived using the T7 primer sets shown in Table S9, using *An. gambiae* cDNA as template.

Mosquitoes were surface sterilized by immersing them in 70% ethanol for 30 seconds and washing them twice in PBS and midguts were dissected in *RNA later* (Invitrogen). Total RNA from mosquito midguts was extracted after homogenization with a pestle motor in *RNA later* using the *RNeasy* kit (QIAGEN). cDNA was synthesized from total RNA using the *QuantiTect Reverse Transcription* kit (QIAGEN).

Quantification of bacterial load or the efficiency of RNAi-mediated silencing was performed using qRT-PCR with the respective primers shown in Table S9. In a 20 µl reaction of *Fast SYBR Green Master Mix* (Applied Biosystems), 1 µl of cDNA template and 2 µl of each respective primer at a 0.5 to 9 µM concentration, optimized for each primer set, were added. The 7500 Real-Time PCR System (Applied Biosystems) was used with its respective software to perform the reaction and any further analysis. The relative abundance of each sample was determined using the standard curve method as described in User Bulletin #2 for the ABI Prism 7700 Sequence Detection system (Applied Biosystems) in which the housekeeping *AgS7* gene was used as an endogenous control.

### 454 pyrosequencing

cDNA pools were amplified with the *GO Taq* DNA polymerase (Promega) using the 16S V4–V6 primers shown in Table S9 and suitable barcode sequences and purified using *PCR purification* and *Gel Extraction* kits (QIAGEN). PCR products were sequenced by Beckman Genomics (Grenoble, France) using the Roche 454 GS FLX+ and standard procedures. The resulting FASTA files were filtered to a minimum read length of 250 bp using *Galaxy* [157] and blasted against the NCBI *16SMicrobial* database using *BLAST+* and *prfectBLAST* [158] with standard *blastn* algorithm settings and 10 maximum target sequences. Further analysis was performed using *MEGAN4* [159].

### Meal size and two-choice preference assays

Sugar meal size was determined through a modified capillary feeder assay [160]. Mosquitoes treated with *LacZ* or *Gr9* dsRNA were antibiotic treated for 5 days, starved overnight and, subsequently, individual mosquitoes were fed on a 5 µl glass capillary (VWR) containing 10% D-(-)-Fructose and 5% v/v scarlet dye. For alive mosquitoes, sugar consumption was determined 16 hours later through the reduction of sugar solution in each capillary. The two-choice preference assay was also conducted based on a previously described capillary feeder assay [160]. Mosquitoes treated with *LacZ* or *Gr9* dsRNA were antibiotic treated for 5 days, starved overnight and placed in pools of 8–11 mosquitoes. Mosquitoes were offered to feed from two capillaries, one containing a sugar solution as above and one also containing *S. marcescens*, prepared as described above for oral infection. Water-containing cotton pads were also used and pools with mosquito mortality were disregarded. 16 hours later, consumption for each capillary was determined based on the reduction of the sugar solution.

## Supporting Information

Figure S1 **Efficacy of antibiotic treatment in reducing the presence of gut bacteria in treated mosquitoes.** *An. gambiae* mosquitoes were antibiotic treated for 5 days with a cocktail of gentamicin, penicillin and streptomycin. Subsequently, 10–15 mosquitoes were surface sterilized and their guts were dissected, homogenized and total RNA was extracted and further used for cDNA synthesis. Mosquitoes kept untreated were processed in the same way. Subsequently, cDNA from antibiotic treated or untreated mosquito pools was used in a qRT-PCR using broad range bacterial 16S primers while *AgS7* primers were used as controls. The bacterial load ±SEM in 3 independent assays, with the qRT-PCR performed at least twice for each assay, can be seen. Asterisks indicate significance, with a p-value<0.0005, in a Mann-Whitney non-parametric test between the bacterial load of untreated and antibiotic treated mosquitoes.
(TIF)

Figure S2 **Efficacy of RNAi-mediated silencing of** ***FN3D1–3*** **,** ***Gr9*** **and** ***NPF*** **.** Mosquitoes were treated with dsRNA targeting the *FN3D1*, *FN3D2*, *FN3D3*, *Gr9* or *NPF* transcripts or with the *dsLacZ* control. The *Gr9* dsRNA targets the 5′ exon 1, which mainly limits the RA and RG splice variants. The relative expression of each transcript in the mosquito gut was determined 5 to 6 days post dsRNA treatment, normalized to the endogenous *AgS7* control, in mosquitoes treated with the respective dsRNA by qRT-PCR and primers targeting the respective transcript in a region not targeted by the respective dsRNA. Silencing efficiency was determined by further normalizing the relative expression of each transcript in mosquitoes treated with the respective dsRNA to the relative expression in the *dsLacZ* treated control. The average ±SEM of relative expression is shown for at least 3 independent assays, with the qRT-PCR performed at least twice for each assay.
(TIF)

Figure S3 ***FN3D1–3*** **silencing modulates total bacteria and** ***Serratia*** **in a non-uniform way in mosquitoes retaining their natural gut microbiota.** Mosquitoes retaining their natural gut microbiota (*Ab−Sm−*) were treated with *FN3D1* (S3A), *FN3D2* (S3B) or *FN3D3* (S3C) dsRNA and bacterial load was normalized to the respective *dsLacZ* treated control. Bacterial load using broad range 16S or *Serratia*-specific primers is shown for 4 independent assays (Replicates 1–4 as indicated above each pair of bars).
(TIF)

Figure S4 **Oral infection with** ***Asaia*** **following** ***FN3D1–3*** **silencing.** Antibiotic treated mosquitoes were orally infected with bacteria of the genus *Asaia* following treatment with *FN3D1–3* dsRNA or the *dsLacZ* control. Bacterial load was determined in the guts of surface sterilized mosquitoes dissected 5 days post infection, using qRT-PCR with 16S broad range bacterial primers and the *AgS7* control. The bacterial load ±SEM in 3 independent assays, with the qRT-PCR reaction performed at least twice for each assay, can be seen.
(TIF)

Figure S5 **Two-choice preference assay between sugar solutions containing or not** ***S. marcescens*** **.** Antibiotic treated mosquitoes treated either with *LacZ* or *Gr9* dsRNA were starved overnight and, subsequently, pools of 8–11 mosquitoes were offered a choice of two meals in separate 5 µl capillaries, one containing a sugar solution containing a dye used to measure consumption and ensure uptake from the mosquitoes and one also containing *S. marcescens*, at the same concentration as used for oral infection. 16 hours later, consumption was measured for each capillary and, for each mosquito pool, the ratio of the % consumption in the *Serratia*-containing capillary to the % consumption in the sugar-only capillary was determined. Overall, the consumption percentage ratio of *Serratia*-containing versus sugar-only capillaries was determined for 6 *LacZ* and 11 *Gr9* dsRNA treated mosquito pools. The average ±SEM percentage ratio for each dsRNA treatment can be seen. Significant differences were assessed using the non-parametric Mann-Whitney test resulting in a p-value of 0.7395, indicated as non-significant (NS). A percentage ratio of <100% would indicate aversion to the *Serratia*-containing solution while a percentage ratio of >100% would indicate attraction to the *Serratia*-containing solution for mosquitoes treated with the respective dsRNA, as indicated.
(TIF)

Figure S6 **Significantly overrepresented GO terms in the set of more than 1.75-fold regulated genes following** ***S. marcescens*** **infection.** A hypergeometric test with Benjamini-Hochberg correction was used to compare the representation of genes corresponding to the same GO term in the set of 97 more than 1.75-fold regulated genes following *S. marcescens* infection to the respective representation in the *An. gambiae* genome, as annotated in the *Pfalcip_Agamb2009* microarray design. 16 GO terms corresponded to significantly overrepresented groups of genes, shown in orange type in the GO directed acyclic graph, with GO terms in the same path that did not meet the p-value cut-off shown in black type. For significantly overrepresented GO terms, the number of corresponding genes in the set of more than 1.75-fold regulated genes is shown in parenthesis. A table with the regulated transcripts corresponding to GO terms at each final leaf node is shown, including, for each transcript, the Transcript ID, an assigned name or description based on *Interpro*-predicted domains or homologies with *Drosophila* counterparts and the observed transcriptional regulation following *S. marcescens* infection.
(TIF)

Table S1 **Complete list of SNP loci assayed for association with the** ***S. marcescens*** **infection phenotype.** SNP loci interrogated in a 400 k Affymetrix SNP genotyping array. The MAF for the highly infected and non-infected phenotypic pools and the calculated MAF difference can be seen for each interrogated SNP locus.
(RAR)

Table S2 **Complete list of 10-SNP loci windows assayed for association with the** ***S. marcescens*** **infection phenotype.** The 40,007 sliding 10-SNP windows assayed for association with the *S. marcescens* infection phenotype are shown along with the genomic position of each 10-SNP window, the calculated average MAF difference and the assigned p-value of the permutation analysis conducted.
(XLSX)

Table S3 **Individual SNP loci associated with the** ***S. marcescens*** **infection phenotype.** A total of 140 out of 400,071 SNP loci that showed MAF difference >0.5 between the highly infected and non-infected *S. marcescens* phenotypic pools are shown. Each column contains for each SNP locus: the chromosome it resides, the MAF for the highly infected and non-infected phenotypic pools and the calculated MAF difference, the genomic position of the SNP locus and the calculated 5 kb radius (position −5 kb and position +5 kb columns), the residing peak for each SNP locus as assigned in Figure 2 and genes found within a 5 kb radius of this SNP locus based on the AgamP3.7 annotation of the *An. gambiae* genome.
(XLSX)

Table S4 **10-SNP loci windows associated with the** ***S. marcescens*** **infection phenotype.** A total of 44 out of 40,007 10-SNP loci windows showed a significant p-value of <10^−5^ in a permutation analysis following Bonferroni correction, when assayed for association with the *S. marcescens* infection phenotype. Each column contains for each 10-SNP locus window: The chromosome it resides, the genomic position (Center-Start-Stop columns), the average MAF difference calculated, the assigned p-value of the permutation analysis, the peak it resides as assigned in Figure 2 and the genes residing within the 10-SNP locus window based on the AgamP3.7 annotation of the *An. gambiae* genome.
(XLSX)

Table S5 **Complete list of** ***An. gambiae*** **genes associated with the outcome** ***of S. marcescens*** **infection.** 138 *An. gambiae* genes were associated with the outcome of *S. marcescens* infection, either by residing within a 5 kb radius of SNP loci with MAF difference >0.5 (SNP) or by residing within 10-SNP loci windows with a <10^−5^ p-value in a permutation analysis (Permutation). For genes identified by both approaches, both SNP and Permutation are noted. For each gene, the Description column includes the assigned name, if any, or a description based on homologs or *Interpro*-predicted domains.
(XLSX)

Table S6 **16S metagenomic profiling in** ***FN3D1–3*** **or** ***LacZ*** **dsRNA treated mosquitoes retaining their natural gut microbiota.** cDNA pools from *Ab−Sm−* mosquitoes following *FN3D1–3* or *LacZ* dsRNA treatment were sequenced using 454 pyrosequencing targeting the V4–V6 region of the bacterial 16S rRNA transcript. Results are shown for each cDNA pool corresponding to a dsRNA treatment shown in Figure 4. In each case, taxonomic identification and alignment of reference sequences for each identified taxon is shown along with the number of sequence reads assigned. For each bacterial genus, the reference sequence with most alignment hits (sequence reads) is shown. For *Serratia*, the reference sequence with most alignment hits is highlighted and was used for categorization between *Serratia* and other *Enterobacteriaceae*.
(XLSX)

Table S7 ***An. gambiae*** **transcripts with more than 1.75-fold regulation following** ***S. marcescens*** **infection.** 99 *An. gambiae* transcripts showing more than 1.75-fold up or down regulation following *S. marcescens* infection. For each transcript, the Transcript ID is shown along with its designated name or functional description, the assigned functional class, the fold-change regulation and the p-values obtained by a t-test against zero using the log2-transformed values for each interrogated probe.
(XLSX)

Table S8 **GO terms significantly overrepresented in the set of more than 1.75-fold regulated genes following** ***S. marcescens*** **infection.** 16 enriched GO terms as determined by a hypergeometric test followed by Benjamini-Hochberg correction using a 0.1 p-value cut-off are shown. The GO ID, accession number and description of each GO term is shown along with the results of the test for each GO term.
(XLSX)

Table S9 **Sequences of primers used for qRT-PCR, dsRNA synthesis or 454 pyrosequencing.** For each forward or reverse primer, its use is indicated along with the respective sequence.
(XLSX)
