# Supplementary figures and images for: Genetic Dissection of Anopheles gambiae Gut Epithelial Responses to Serratia marcescens
Source: PLoS Pathog. 2014 Mar 6;10(3):e1003897. doi: 10.1371/journal.ppat.1003897 (PMC3946313; doi:10.1371/journal.ppat.1003897)

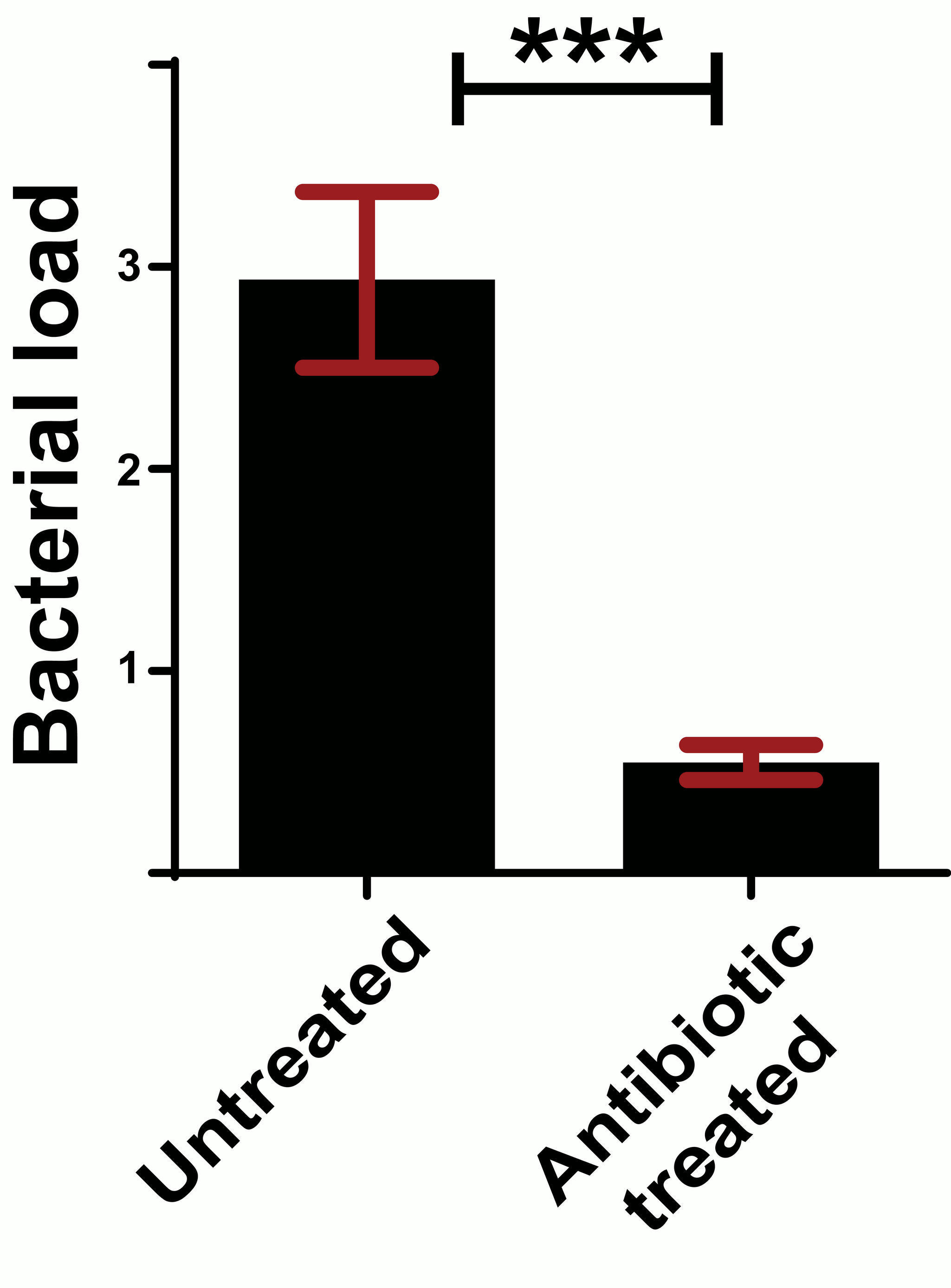

Supplement: Figure S1 — Efficacy of antibiotic treatment in reducing the presence of gut bacteria in treated mosquitoes. An. gambiae mosquitoes were antibiotic treated for 5 days with a cocktail of gentamicin, penicillin and streptomycin. Subsequently, 10–15 mosquitoes were surface sterilized and their guts were dissected, homogenized and total RNA was extracted and further used for cDNA synthesis. Mosquitoes kept untreated were processed in the same way. Subsequently, cDNA from antibiotic treated or untreated mosquito pools was used in a qRT-PCR using broad range bacterial 16S primers while AgS7 primers were used as controls. The bacterial load ±SEM in 3 independent assays, with the qRT-PCR performed at least twice for each assay, can be seen. Asterisks indicate significance, with a p-value<0.0005, in a Mann-Whitney non-parametric test between the bacterial load of untreated and antibiotic treated mosquitoes. (TIF) [file ppat.1003897.s001.tif]

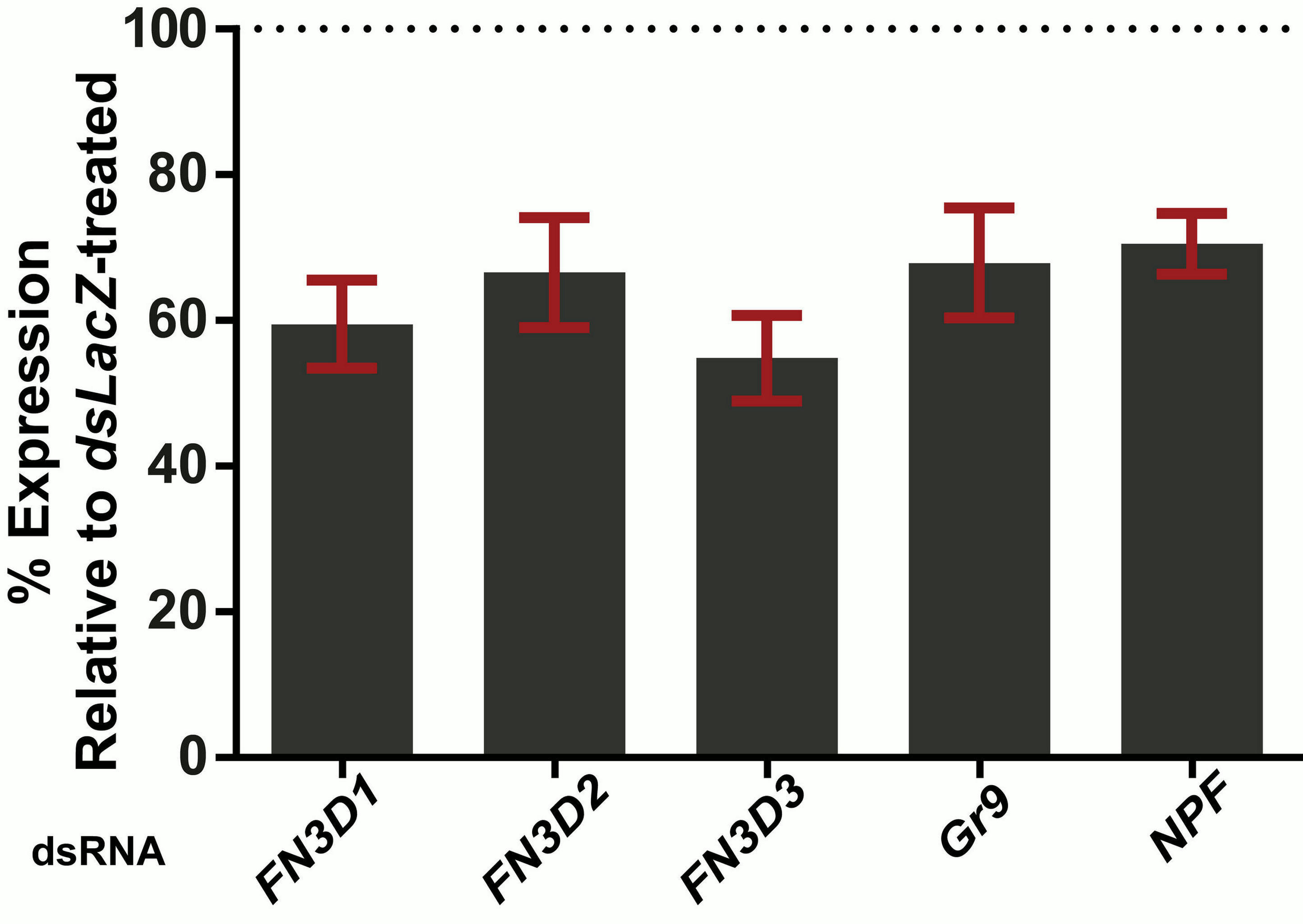

Supplement: Figure S2 — Efficacy of RNAi-mediated silencing of FN3D1–3 , Gr9 and NPF . Mosquitoes were treated with dsRNA targeting the FN3D1, FN3D2, FN3D3, Gr9 or NPF transcripts or with the dsLacZ control. The Gr9 dsRNA targets the 5′ exon 1, which mainly limits the RA and RG splice variants. The relative expression of each transcript in the mosquito gut was determined 5 to 6 days post dsRNA treatment, normalized to the endogenous AgS7 control, in mosquitoes treated with the respective dsRNA by qRT-PCR and primers targeting the respective transcript in a region not targeted by the respective dsRNA. Silencing efficiency was determined by further normalizing the relative expression of each transcript in mosquitoes treated with the respective dsRNA to the relative expression in the dsLacZ treated control. The average ±SEM of relative expression is shown for at least 3 independent assays, with the qRT-PCR performed at least twice for each assay. (TIF) [file ppat.1003897.s002.tif]

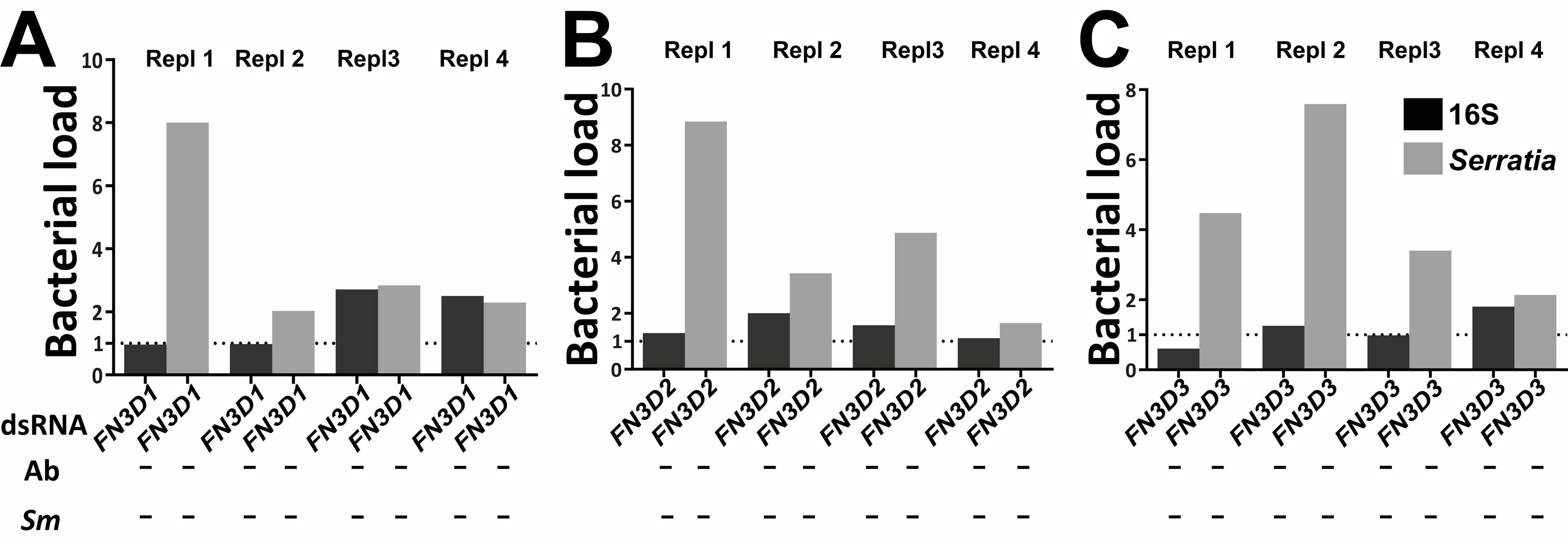

Supplement: Figure S3 — FN3D1–3 silencing modulates total bacteria and Serratia in a non-uniform way in mosquitoes retaining their natural gut microbiota. Mosquitoes retaining their natural gut microbiota (Ab−Sm−) were treated with FN3D1 (S3A), FN3D2 (S3B) or FN3D3 (S3C) dsRNA and bacterial load was normalized to the respective dsLacZ treated control. Bacterial load using broad range 16S or Serratia-specific primers is shown for 4 independent assays (Replicates 1–4 as indicated above each pair of bars). (TIF) [file ppat.1003897.s003.tif]

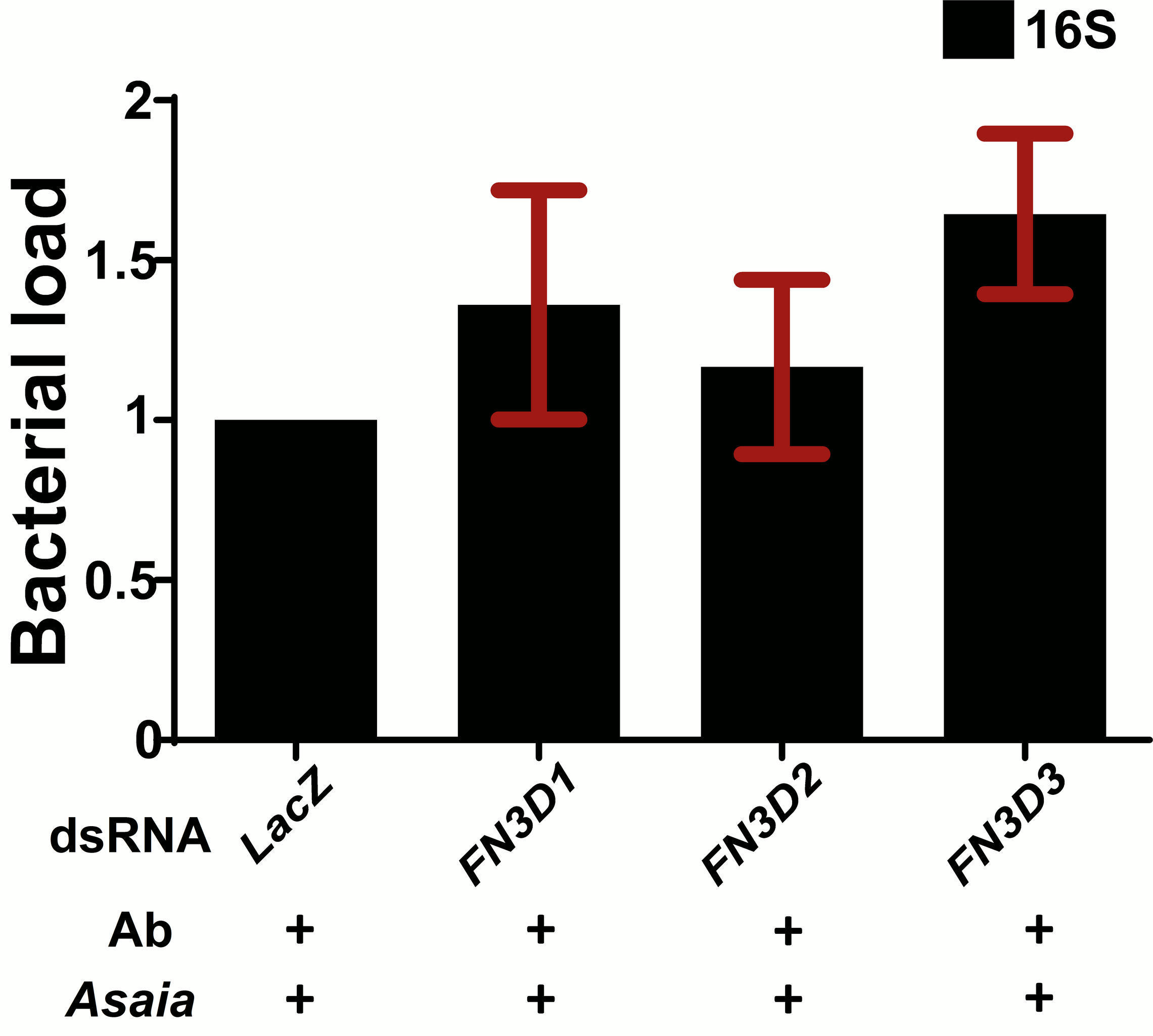

Supplement: Figure S4 — Oral infection with Asaia following FN3D1–3 silencing. Antibiotic treated mosquitoes were orally infected with bacteria of the genus Asaia following treatment with FN3D1–3 dsRNA or the dsLacZ control. Bacterial load was determined in the guts of surface sterilized mosquitoes dissected 5 days post infection, using qRT-PCR with 16S broad range bacterial primers and the AgS7 control. The bacterial load ±SEM in 3 independent assays, with the qRT-PCR reaction performed at least twice for each assay, can be seen. (TIF) [file ppat.1003897.s004.tif]

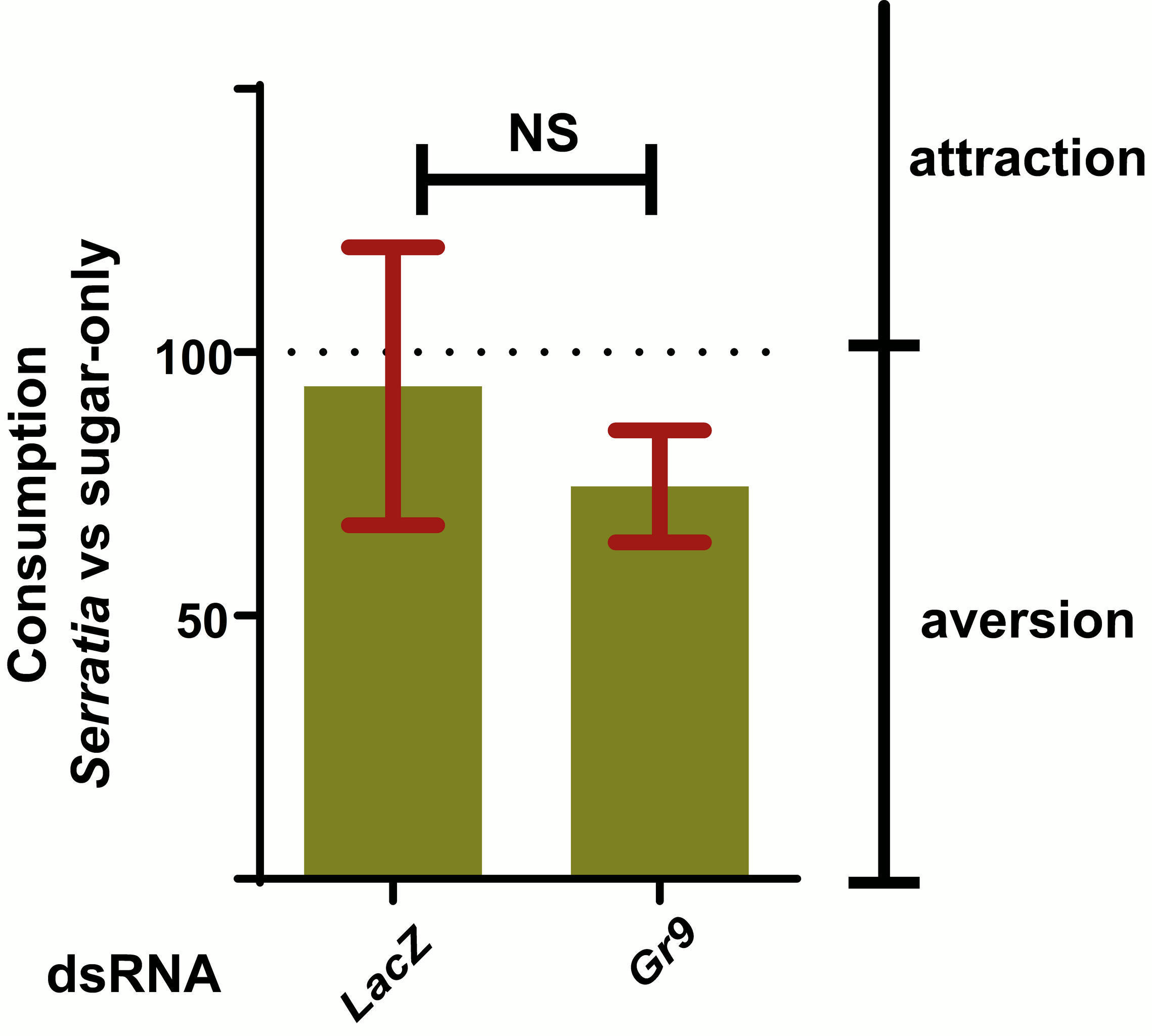

Supplement: Figure S5 — Two-choice preference assay between sugar solutions containing or not S. marcescens . Antibiotic treated mosquitoes treated either with LacZ or Gr9 dsRNA were starved overnight and, subsequently, pools of 8–11 mosquitoes were offered a choice of two meals in separate 5 µl capillaries, one containing a sugar solution containing a dye used to measure consumption and ensure uptake from the mosquitoes and one also containing S. marcescens, at the same concentration as used for oral infection. 16 hours later, consumption was measured for each capillary and, for each mosquito pool, the ratio of the % consumption in the Serratia-containing capillary to the % consumption in the sugar-only capillary was determined. Overall, the consumption percentage ratio of Serratia-containing versus sugar-only capillaries was determined for 6 LacZ and 11 Gr9 dsRNA treated mosquito pools. The average ±SEM percentage ratio for each dsRNA treatment can be seen. Significant differences were assessed using the non-parametric Mann-Whitney test resulting in a p-value of 0.7395, indicated as non-significant (NS). A percentage ratio of <100% would indicate aversion to the Serratia-containing solution while a percentage ratio of >100% would indicate attraction to the Serratia-containing solution for mosquitoes treated with the respective dsRNA, as indicated. (TIF) [file ppat.1003897.s005.tif]

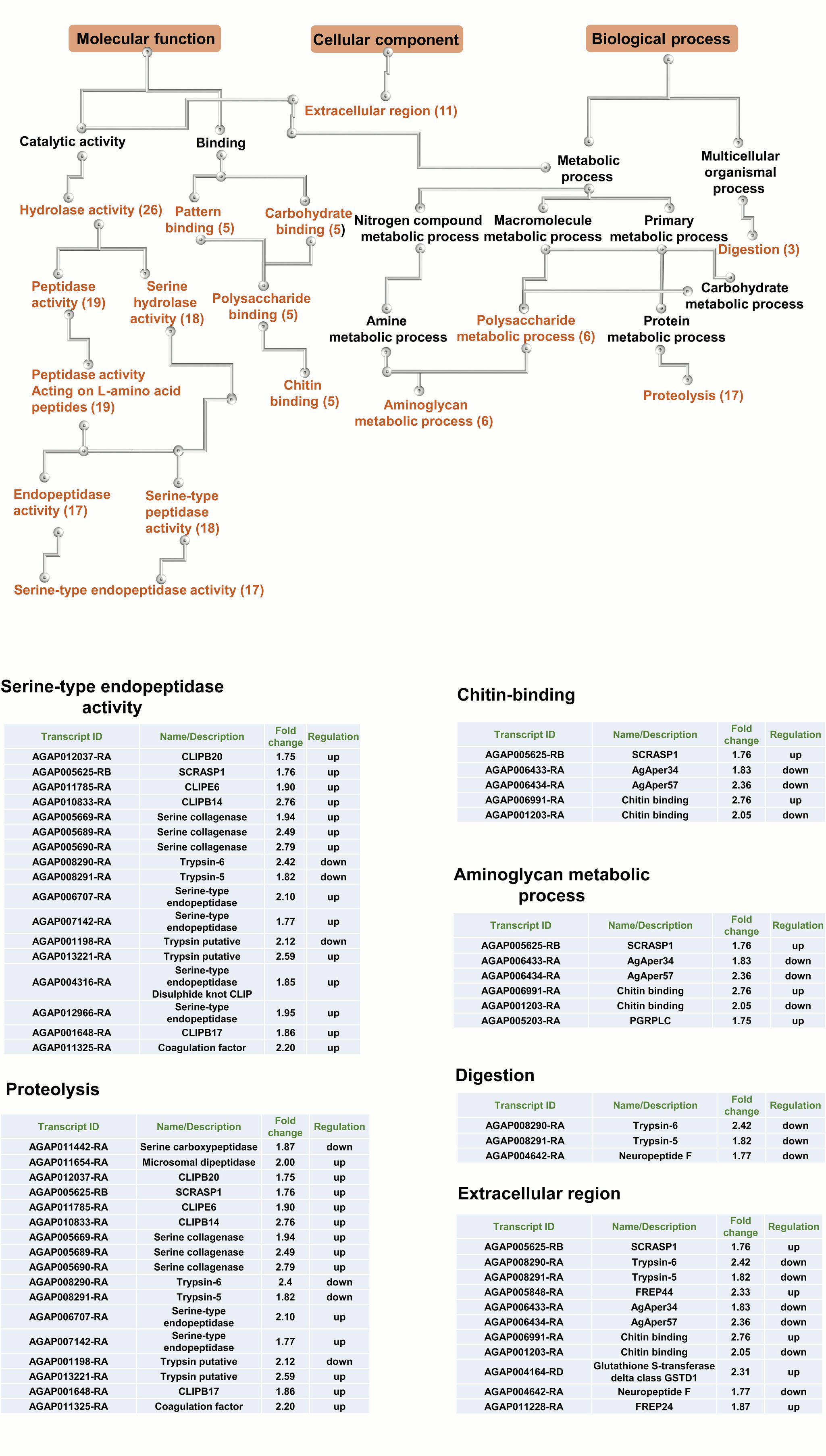

Supplement: Figure S6 — Significantly overrepresented GO terms in the set of more than 1.75-fold regulated genes following S. marcescens infection. A hypergeometric test with Benjamini-Hochberg correction was used to compare the representation of genes corresponding to the same GO term in the set of 97 more than 1.75-fold regulated genes following S. marcescens infection to the respective representation in the An. gambiae genome, as annotated in the Pfalcip_Agamb2009 microarray design. 16 GO terms corresponded to significantly overrepresented groups of genes, shown in orange type in the GO directed acyclic graph, with GO terms in the same path that did not meet the p-value cut-off shown in black type. For significantly overrepresented GO terms, the number of corresponding genes in the set of more than 1.75-fold regulated genes is shown in parenthesis. A table with the regulated transcripts corresponding to GO terms at each final leaf node is shown, including, for each transcript, the Transcript ID, an assigned name or description based on Interpro-predicted domains or homologies with Drosophila counterparts and the observed transcriptional regulation following S. marcescens infection. (TIF) [file ppat.1003897.s006.tif]
